# The MprF homolog LysX synthesizes lysyl-diacylglycerol contributing to antibiotic resistance and virulence

**DOI:** 10.1128/spectrum.01429-23

**Published:** 2023-09-28

**Authors:** Cameron P. Gill, Christopher Phan, Vivien Platt, Danielle Worrell, Thomas Andl, Hervé Roy

**Affiliations:** 1 Burnett School of Biomedical Sciences, College of Medicine, University of Central Florida, Orlando, Florida, USA; University of Guelph, College of Biological Science, Guelph, Ontario, Canada

**Keywords:** tRNA, diacylglycerol, *Corynebacterium*, *Mycobacterium*, antibiotic resistance

## Abstract

Lysyl-diacylglycerol (Lys-DAG) was identified three decades ago in *Mycobacterium phlei*, but the biosynthetic pathway and function of this aminoacylated lipid have since remained uncharacterized. Combining genetic methods, mass spectrometry, and biochemical approaches, we show that the multiple peptide resistance factor (MprF) homolog LysX from *Corynebacterium pseudotuberculosis* and two mycobacterial species is responsible for Lys-DAG synthesis. LysX is conserved in most Actinobacteria and was previously implicated in the synthesis of another modified lipid, lysyl-phosphatidylglycerol (Lys-PG), in *Mycobacterium tuberculosis*. Although we detected low levels of Lys-PG in the membrane of *C. pseudotuberculosis*, our data suggest that Lys-PG is not directly synthesized by LysX and may require an additional downstream pathway, which is as yet undefined. Our results show that LysX in *C. pseudotuberculosis* is a major factor of resistance against a variety of positively charged antibacterial agents, including cationic antimicrobial peptides (e.g., human peptide LL-37 and polymyxin B) and aminoglycosides (e.g., gentamycin and apramycin). Deletion of *lysX* caused an increase in cellular membrane permeability without dissipation of the membrane potential, suggesting that loss of the protein does not result in mechanical damage to the cell membrane. Furthermore, *lysX*-deficient cells exhibited an attenuated virulence phenotype in a *Galleria mellonella* infection model, supporting a role for LysX during infection. Altogether, Lys-DAG represents a novel molecular determinant for antimicrobial resistance and virulence that may be widespread in Actinobacteria and points to a richer landscape than previously realized of lipid components contributing to overall membrane physiology in this important bacterial phylum.

**IMPORTANCE:**

In the past two decades, tRNA-dependent modification of membrane phosphatidylglycerol has been implicated in altering the biochemical properties of the cell surface, thereby enhancing the antimicrobial resistance and virulence of various bacterial pathogens. Here, we show that in several Actinobacteria, the multifunctional protein LysX attaches lysine to diacylglycerol instead of phosphatidylglycerol. We found that lysyl-diacylglycerol (Lys-DAG) confers high levels of resistance against various cationic antimicrobial peptides and aminoglycosides and also enhances virulence. Our data show that Lys-DAG is a lipid commonly found in important actinobacterial pathogens, including *Mycobacterium* and *Corynebacterium* species.

## INTRODUCTION

The cell envelope is the first line of defense that protects bacteria from the stressors of the environment. Cells have evolved systems to adapt the constituents of their cellular envelope in response to changing environmental conditions (1). One of these systems utilizes aminoacyl-tRNA (aa-tRNA) as a donor of amino acids (aa’s) for the aminoacylation of the polar head groups of membrane lipids. The multiple peptide resistance factor (MprF), first discovered in *Staphylococcus aureus*, exhibits lysyl-phosphatidylglycerol synthase (LysPGS) activity that uses Lys-tRNA to aminoacylate the free hydroxyl group of phosphatidylglycerol (PG) to produce lysyl-phosphatidylglycerol (Lys-PG). The addition of Lys to membrane PG decreases the net negative charge of the cell surface, thereby increasing resistance to various cationic antimicrobial peptides (CAMPs) and enhancing bacterial evasion of the killing activities of neutrophils and macrophages (2). MprF is a bi-functional integral membrane protein comprising a GCN5-related N-acetyltransferase (GNAT)-like C-terminal domain supporting the aa-tRNA/phosphatidylglycerol transferase activity and an N-terminal flippase (i.e., translocase) domain, which flips neosynthesized lipids from the inner surface of the cytoplasmic membrane to the outer surface (3, 4).

Since the initial discovery of MprF in *S. aureus*, a family of orthologs has been uncovered, with members appearing in the genomes of most bacterial phyla, in archaea (5), and, most recently, in fungal pathogens (6 – 8). Members of this family of proteins exhibit differing specificities for both the aa’s and lipids that are used as substrates. Lipid modification by Lys and Ala (9, 10) has been described in various bacterial species, while Gly and Asp are substrates used by the fungal proteins (6 – 8). Likewise, some of these enzymes act on lipids that are distinct from PG, such as cardiolipin in proteobacteria (11, 12) and diacylglycerol (DAG) in *Corynebacterium glutamicum* (1 3). The more distant MprF orthologs found in fungi aminoacylate a sterol (i.e., ergosterol) instead of a glycerolipid (e.g., PG, DAG, or cardiolipin) (6 – 8, 14), demonstrating the substrate diversity among this family of proteins.

The phenotypes associated with Lys-PG are by far the best characterized. It is thought that lysylation of PG decreases the net electrostatic charge of the cytoplasmic membrane, thereby enhancing resistance to antibiotics and CAMPs, substances secreted by the innate immune system of infected hosts [for review, see references (15 – 18)]. Lys-PG increases bacterial virulence in cell cultures and animal models (11, 19, 20) and enhances resistance to both macrophages (20) and neutrophils (2, 21). These effects have been observed in the context of a variety of Gram positive human pathogens such as *Staphylococcus aureus* (2, 19, 21, 22), *Bacillus anthracis* (23), *Listeria monocytogenes* (11, 24, 25), and *Mycobacterium tuberculosis* (20). Similar effects have been described in Gram negative species such as *Pseudomonas aeruginosa* (5, 9, 26) and *Rhizobium tropici* (27).

MprF homologs in Actinobacteria are structurally and functionally distinct from the canonical proteins found in other bacterial species. For instance, an alanyl-diacylglycerol synthase (AlaDAGS) responsible for the synthesis of alanyl-diacylglycerol (Ala-DAG) was identified in *Corynebacterium glutamicum* (13). In *Mycobacterium turberculosis*, the gene *lysX* (Rv1640c) encodes a 1,176-amino acid long peptide consisting of an integral membrane domain and GNAT-like domain, characteristic of the MprF family of proteins, in addition to a C-terminal lysyl-tRNA synthetase (LysRS) domain used for synthesis of the Lys-tRNA^Lys^ necessary for formation of Lys-PG (20). LysX has been implicated as an important factor for antibiotic resistance and host-pathogen interactions in the *M. tuberculosis* pathogen. Cells harboring mutations in the protein are sensitive to various cationic antibiotics and peptides (20, 28) and show defective growth phenotypes in mouse and guinea pig lung models (20, 29). LysX was also shown to be important for bacterial survival in human monocytes and macrophages (30, 31).

Many Actinobacteria, notably species of the *Corynebacterium*, *Mycobacterium*, and *Nocardia* (CMN) group, exhibit two MprF paralogs (13), one homologous to *C. glutamicum* AlaDAGS and the other to *M. turberculosis* LysX. Here, we study the role of these paralogs in the model organism *Corynebacterium pseudotuberculosis*, an important pathogen responsible for caseous lymphadenitis and other chronic diseases in livestock animals and, occasionally, humans (32, 33). Using a combination of genetic, biochemical, and mass spectrometry (MS) approaches, we show that the LysX homolog in *C. pseudotuberculosis* is responsible for the synthesis of the modified lipid lysyl-diacylglycerol (Lys-DAG). Although Lys-DAG was first reported over three decades ago in *Mycobacterium phlei*, its role and biosynthetic pathway have since remained elusive (34, 35). Lys-DAG was the only amine-containing lipid (ACL) detectable by TLC (thin layer chromatography) in *C. pseudotuberculosis* membrane extracts; however, trace amounts of Lys-PG were also revealed by liquid chromatography-tandem mass spectrometry (LC-MS/MS). To further explore the role of LysX in related organisms, the protein’s activity was interrogated in two additional mycobacterial species. We find that LysX homologs from *Mycobacterium abscessus* and *Mycobacterium parafinicum* also synthesize Lys-DAG. Our data suggest that the only product of LysX activity is Lys-DAG and the low amounts of Lys-PG in *C. pseudotuberculosis* may be the product of a secondary pathway that may use Lys-DAG as a substrate. We show that LysX in *C. pseudotuberculosis* is an important resistance factor against various cationic antibacterial agents, including the human pore-forming peptide LL-37, polymyxin B, and several positively charged aminoglycosides. Deletion of *lysX* disturbs cellular uptake of the cationic dye propidium iodine (PI) without dissipation of the membrane potential, demonstrating that *lysX* is essential for membrane impermeability to cationic compounds but not for maintaining a proton gradient. The *lysX* mutant strain also exhibits an attenuated virulence phenotype in a *Galleria mellonella* infection model.

## RESULTS

### MprF homologs from *C. pseudotuberculosis* synthesize two amine-containing lipids

**FIG 1:**
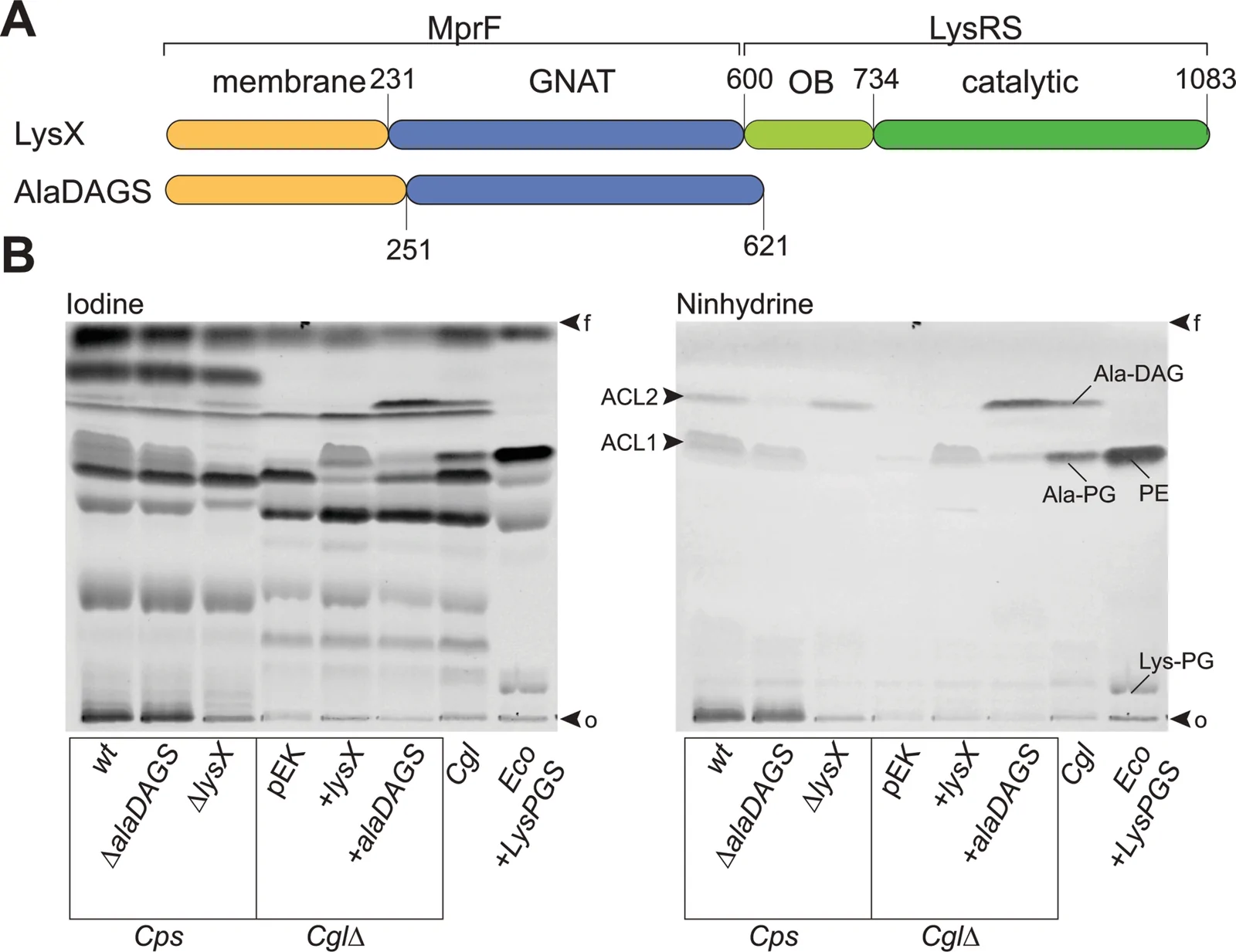
MprF paralogs and analysis of ACLs in *C. pseudotuberculosis*. (**A**) Architecture of MprF paralogs in *C. pseudotuberculosis*. The boundaries of the membrane domain, the GNAT transferase domain, and the LysRS domain were established using known structures of MprF homologs (36, 37, 39). LysX contains features characteristic of a class II LysRS, including an anticodon binding domain (OB-Fold) and an aa-tRNA catalytic domain. (**B**) TLC analysis of lipids from *C. pseudotuberculosis*. Total lipids were visualized using iodine vapors (left image), and ACLs were specifically revealed by spraying the same TLC with ninhydrin reagent (right image). Lipids from wt *C. pseudotuberculosis* (*Cps*), Δ*alaDAGS*, and Δ*lysX* strains are shown in the three left-most lanes in both images. The three next lanes show lipids isolated from *C. glutamicum ΔpesTΔalaDAGS* cells (*Cgl*Δ) (13), harboring either the expression vector pEKEx2 (pEK) alone or heterologously expressing *alaDAGS* or *lysX* from *C. pseudotuberculosis*. Control lipids, Ala-PG, Lys-PG, Ala-DAG, and PE, were obtained from *C. glutamicum* (*Cgl*) and *E. coli* C41, expressing the LysPGS from *C. perfringens* (10), and are shown in the two right-most lanes. ACL1 and 2, amine-containing lipids; o, TLC origin; f, TLC front.

The two MprF homologs from *C. pseudotuberculosis* share a high percentage of identity with the previously characterized LysX from *M. turberculosis* (42%) and AlaDAGS from *C. glutamicum* (45%). Both enzymes exhibit an integral membrane domain with six predicted transmembrane helices (based on the TOPCONS web server) and a GNAT-like domain, which catalyzes the aa-tRNA lipid transferase reaction (Fig. 1A). Both of these domains are characteristic of the MprF family of enzymes (18, 36 – 38). The LysX proteins from *M. turberculosis* and *C. pseudotuberculosis* share other architectural features as well. They both include a LysRS domain at their C-terminal ends. This domain includes a catalytic domain characteristic of a LysRS and an oligonucleotide-binding (OB) fold, which, in the context of a cytosolic LysRS, is used to bind the tRNA anticodon (39) (Fig. 1A). In *M. tuberculosis*, it was demonstrated that the LysRS domain of LysX synthesizes the Lys-tRNA^Lys^ substrate used for Lys-PG formation, which is catalyzed in a secondary reaction by the MprF GNAT-like domain (20).

To determine whether the activities of the *C. pseudotuberculosis* enzymes are identical to the previously characterized proteins, we carried out thin layer chromatography (TLC) analysis of total lipid extracts. Extracted lipids from *C. pseudotuberculosis* revealed two amine-containing lipids (ACL1 and ACL2; Fig. 1B). ACL1 migrated similarly to the control lipids alanyl-phosphatidylglycerol (Ala-PG) and phosphatidylethanolamine (PE), while ACL2 migrated like Ala-DAG. Lipids from Δ*alaDAGS* and Δ*lysX* strains were analyzed to determine which MprF homolog is responsible for the synthesis of each of the ACLs. The Δ*lysX* strain was unable to produce ACL1, and the Δ*alaDAGS* strain was unable to produce ACL2.

To confirm the correlation of the *C. pseudotuberculosis* homologs with the formation of ACL1 and ACL2, LysX and AlaDAGS from *C. pseudotuberculosis* were cloned into the vector pEKEx-2 and expressed heterologously in *C. glutamicum*. The *C. glutamicum* strain Δ*pesT*Δ*alaDAGS* (deprived of the gene associated with the synthesis of Ala-DAG and Ala-PG) was used to avoid interference with the activities of the *C. pseudotuberculosis* enzymes. Expression of *C. pseudotuberculosis lysX* in *C. glutamicum* promoted synthesis of ACL1, while expression of the AlaDAGS homolog promoted synthesis of ACL2 (Fig. 1B). Notably, expression of *lysX* in *C. glutamicum* did not promote the synthesis of Lys-PG, consistent with the results obtained in *C. pseudotuberculosis* described above. Collectively, these data suggest that the AlaDAGS homolog is responsible for the synthesis of Ala-DAG, while LysX mediates the formation of an amine-containing lipid, ACL1, which shares similar chromatographic properties with Ala-PG or PE but not Lys-PG. ACL1 and ACL2 were found to be the main amine-containing lipids produced in wild-type (wt) *C. pseudotuberculosis*.

### LysX catalyzes the tRNA-dependent lysylation of ACL1

**FIG 2:**
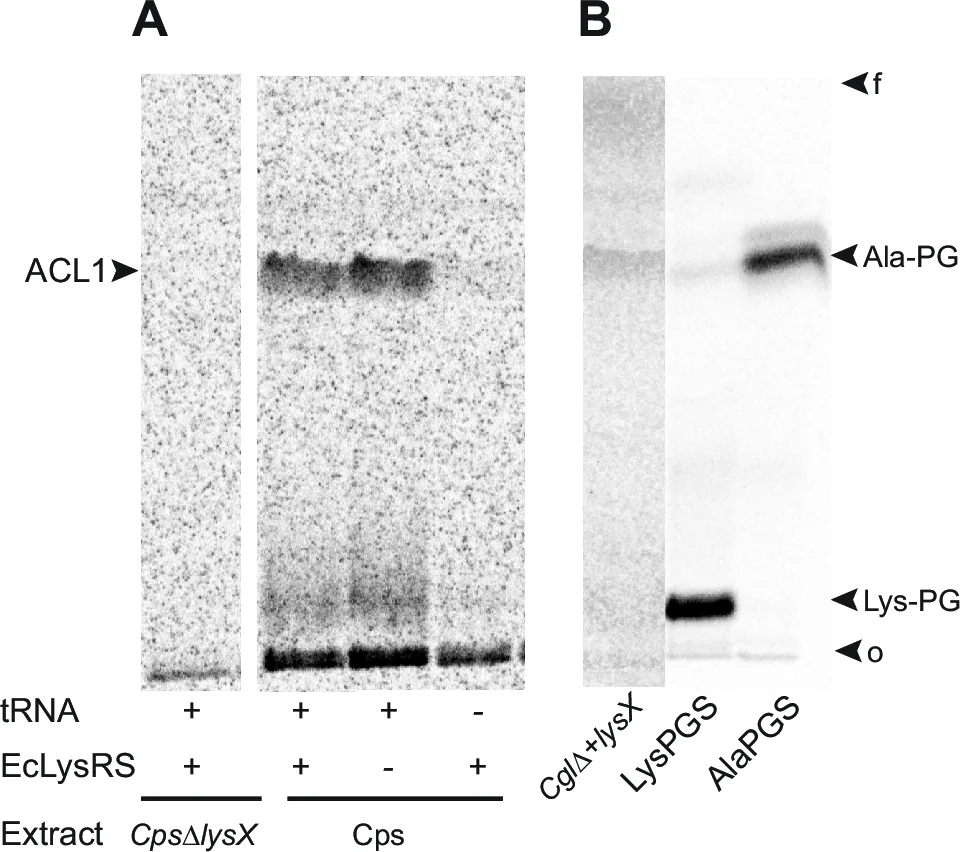
Activity of *C. pseudotuberculosis* LysX *in vitro*. (**A**) LysX activity was reconstituted *in vitro* using [^14^C]-Lys and membrane extracts from *C. pseudotuberculosis* wt (Cps) or Δ*lysX* (CpsΔ*lysX*) cells. The reaction medium was supplemented with DAG and PG in the presence or absence of total tRNAs and LysRS from *E. coli* (EcLysRS), to interrogate the specificity of the reaction. [^14^C]-lysylated products were separated by TLC and visualized by autoradiography. (**B**) Control lanes. ACL1 was obtained from strain *C. glutamicum* heterologously expressing LysX from *C. pseudotuberculosis* and was revealed by spraying with ninhydrin reagent (Fig. 1). [^14^C]-Lys-PG and [^14^C]-Ala-PG were obtained from an *in vitro* reaction medium including membrane extracts from *E. coli* expressing the AlaPGS or LysPGS from *Clostridium perfringens* and the corresponding [^14^C]-Ala and [^14^C]-Lys, respectively (10). TLC front (f) and origin (o) are indicated.

To further define the substrates of LysX and the identity of ACL1, membrane extracts from wild-type *C. pseudotuberculosis* and the Δ*lysX* strain were assayed *in vitro* using [^14^C]-Lys in the presence or absence of total tRNA and LysRS from *Escherichia coli*. Figure 2 shows that no lipid lysylation was observed with membrane extracts from the Δ*lysX* strain. However, [^14^C]-Lys-labeled ACL1 was the main product of the reaction when a membrane extract from the wild-type strain was used. A minor product migrating at the same location as the control Lys-PG (above the TLC origin; Fig. 2) was also observed, suggesting that Lys-PG synthase activity may also be present in the membrane extract from *C. pseudotuberculosis*. It is worth noting that some radiolabeled material did not migrate and was detected at the origin of the TLC. Production of both ACL1 and the minor product was dependent on the addition of tRNA, as the absence of tRNA abolished their synthesis. ACL1 synthesis was independent, however, of exogenous LysRS, suggesting that the LysRS domain of LysX is functional in the assay and able to aminoacylate the tRNA used for lipid labeling. Altogether, these results suggest that *C. pseudotuberculosis* LysX, like its homolog in *M. tuberculosis*, utilizes lysine for lipid aminoacylation. However, contrary to the case in *M. tuberculosis*, the predominant recipient of lysine in the *C. pseudotuberculosis* LysX pathway appears to be a lipid other than PG.

### Identification of *C. pseudotuberculosis* ACLs by LC-MS/MS

**FIG 3:**
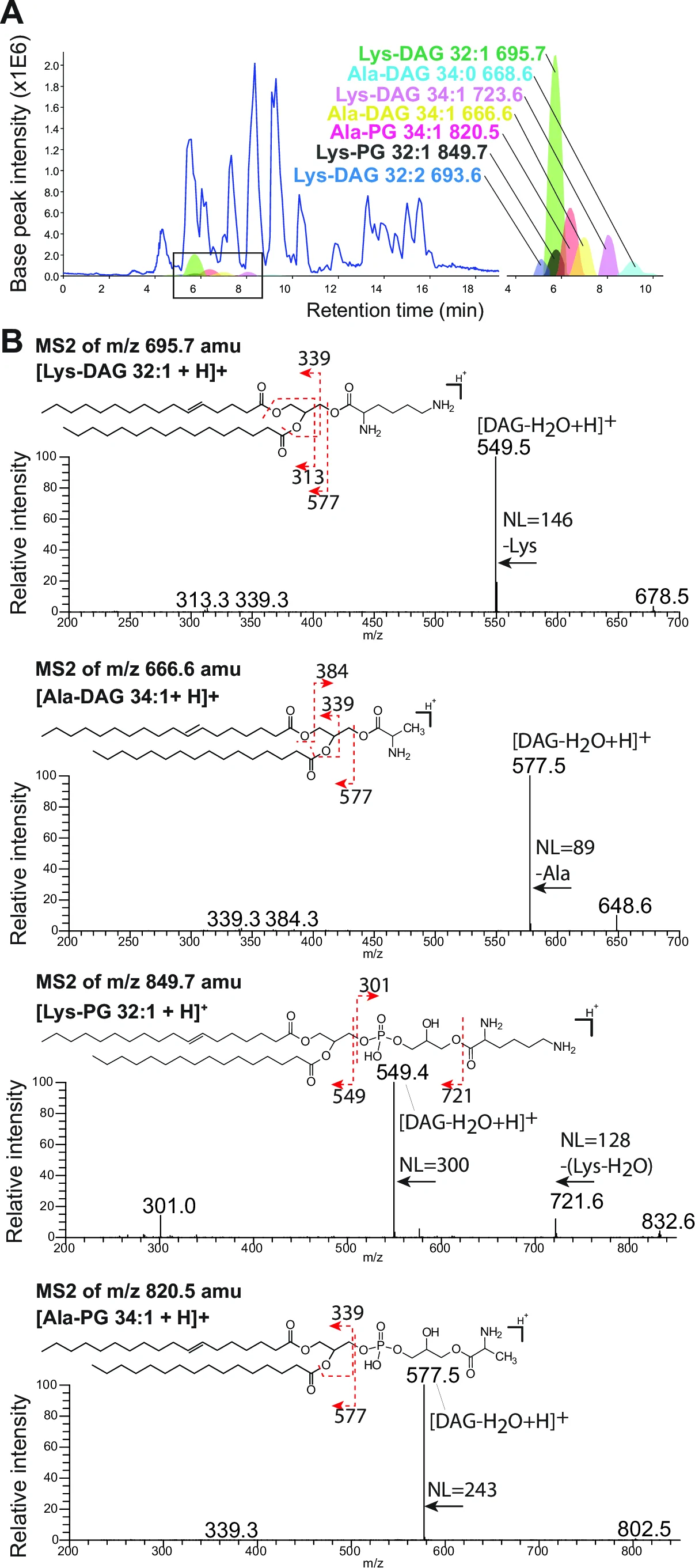
LC-MS/MS analysis of aminoacylated lipids in *C. pseudotuberculosis*. (**A**) Base peak (m/z 200–2000) chromatogram of total lipids (left) and chromatographic deconvolution of the different species of aminoacylated PG and DAG (right). (**B**) Collision-induced dissociation of MS1 peaks at m/z 695, 666, 849, and 820, corresponding to Lys-DAG, Ala-DAG, Lys-PG, and Ala-PG, respectively. All data were acquired in the positive mode.

To determine the structure of ACL1, lipid extracts from wild-type *C. pseudotuberculosis* and deletion strains (Δ*lysX* and Δ*alaDAGS*) were analyzed by LC-MS/MS set to the positive mode. Four aminoacylated lipids were identified in the lipid extract from the wild-type strain. Ala-PG (34:1) and Lys-PG (32:1) were identified, as well as various species of Ala-DAG (34:0, 34:1) and Lys-DAG (32:1, 32:2, 34:1; Fig. 3). The predominant MS2 ion products corresponded to those of a protonated, dehydrated diacylglycerol moiety ([DAG-H_2_O + H]^+^; Fig. 3B) (40). Ala-PG and Ala-DAG exhibited fragmentation patterns identical to those previously reported, with neutral losses of 243 and 89 amu, corresponding to the loss of the alanylated polar head groups of these lipids (alanyl-glycerophosphate and ala, respectively) (13). Identical fragmentation patterns with neutral losses corresponding to the polar head groups of Lys-PG and Lys-DAG (300 amu for lysyl-glycerophosphate and 146 amu for Lys) were also observed. A protonated ion fragment corresponding to the lysyl-glycerophosphate polar head moiety was also detected at 301 amu, consistent with previous observations for Lys-PG (23). Ala-PG and Ala-DAG were absent from lipids extracted from Δ*alaDAGS* cells, and Lys-DAG was missing from the Δ*lysX* strain (data not shown), further supporting the identity of these lipids and their associations with the corresponding enzymes. The LC-MS/MS chromatogram suggests that Lys-DAG is the main lysylated lipid in wild-type *C. pseudotuberculosis*, consistent with the TLC analysis of membrane lipids described above (Fig. 1). LC-MS/MS analysis of lipids isolated from *C. glutamicum* cells heterologously expressing *lysX* revealed several species of Lys-DAG (32:1, 34:1, 34:2, 36:1, 36:2), but no Lys-PG was detected (data not shown). These data suggest that Lys-DAG is the unique product of LysX in this species. Lys-PG may be produced by a secondary enzyme in a fashion similar to the pathway used for the synthesis of Ala-PG in *C. glutamicum* (13).

### Confirmation of Lys-DAG by isotopic labeling

**FIG 4:**
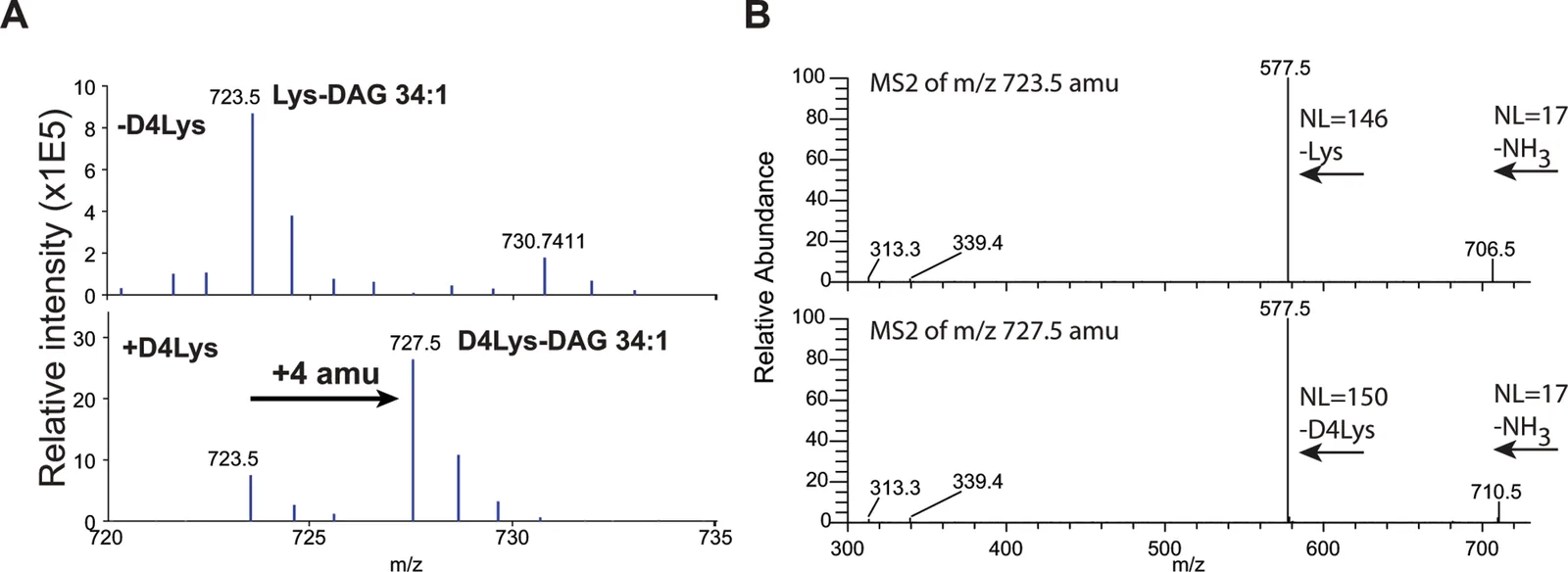
Isotopic labeling of Lys-DAG *in vitro*. (**A**) *C. pseudotuberculosis* membrane extract was used in a tRNA-dependent lipid aminoacylation assay in the absence (top) or presence of D4Lys (bottom). (**B**) MS2 analysis of MS1 peaks at m/z 723 (Lys-DAG 34:1) and 727 (D4Lys-DAG 34:1).

To our knowledge, the neutral losses described above, together with the observation of dehydrated DAG as an ion product (i.e., [DAG + H-H_2_O]^+^ in Fig. 3), are not consistent with any other known lipids (40, 41), lending support to our determination of Lys-DAG as the product of LysX activity in *C. pseudotuberculosis*. However, the neutral loss of 146 amu, consistent with the loss of Lys, could also correspond to the loss of a rhamnosyl group, even though no known rhamnosylated lipids would yield [DAG + H-H_2_O]^+^ as an ion product (42 – 44). Nevertheless, to confirm the identity of Lys-DAG in the system, isotopic labeling was performed *in vitro* using a membrane extract from *C. pseudotuberculosis* as a source of LysX, in the presence or absence of tetradeuterated L-Lys (D4Lys, Fig. 4), and with supplemental PG and DAG added to the reaction. The products of the reaction were analyzed by MS2. In the absence of D4Lys, a peak corresponding to Lys-DAG 34:1, which was pre-existing in the membrane extract, was detected at a m/z of 723, while the addition of D4Lys promoted synthesis of the labeled D4Lys-DAG 34:1 at a m/z value of 727.5. MS2 analysis of each MS1 peak demonstrated characteristic fragmentation of Lys-DAG 34:1, with a neutral loss of 150 amu for the loss of tetradeuterated Lys and 146 amu for the loss of the unlabeled amino acid. Altogether, these data confirm the identity of ACL1 as Lys-DAG and that this lipid is synthesized by LysX.

### LysX from *M. abscessus* and *M. parafinicum* produces Lys-DAG

Previous reports suggested that LysX in *M. tuberculosis* synthesizes Lys-PG instead of Lys-DAG (20, 29, 30). Based on our experimental evidence regarding the role of LysX in the synthesis of Lys-DAG and the observation that this lipid occurs in other mycobacterial species (i.e., *M. phlei*) (34), we decided to investigate LysX-mediated lipid aminoacylation in two additional mycobacterial species. LysX from *M. parafinicum* was cloned into pEKEx-2 and heterologously expressed in *C. glutamicum* lacking endogenous MprF homologs (i.e., the Δ*pesT*Δ*alaDAGS* strain). LC-MS/MS analysis of lysylated lipids isolated from LysX-expressing cells revealed the presence of Lys-DAG 34:1 but not of Lys-PG (Fig. S1). To investigate the role of LysX in *M. abscessus*, the *lysX* gene was disrupted using the strategy developed by Viljoen et al. (45) to generate the knockout strain, *lysX*:pVT. LC-MS/MS analysis of lipids from wild-type *M. abscessus* revealed the presence of several species of Lys-DAG (32:0, 32:1, 34:0, 34:1, 36:1) but not Lys-PG. As expected, Lys-DAG was entirely absent from the *lysX*:pVT strain (data not shown). These results confirm that the product of LysX aminoacylation in these two mycobacterial species is Lys-DAG and not Lys-PG.

### LysX increases resistance to positively charged antibiotics

Lysylation of PG or CL was reported to increase antimicrobial resistance against various CAMPs and other classes of antimicrobials such as penicillins and aminoglycosides. These observations were made in diverse bacterial genera such as *Staphylococcus* (2, 46, 47), *Listeria* (11), *Bacillus* (23), and *Mycobacterium* (20). To investigate the role of LysX-mediated lysylation in *C. pseudotuberculosis*, we first assessed the impact of *lysX* mutations on bacterial generation time (Fig. S2). Δ*lysX* cells grew significantly slower (by 46%) than the wild-type strain (*P* < 0.0001), while Δ*alaDAGS* cells were unaffected. To determine the effect of lipid aminoacylation on the antibiotic resistance of *C. pseudotuberculosis*, minimum inhibitory concentrations (MICs) of various inhibitors were measured for the wild-type and mutant strains. Disruption of *lysX* decreased the MIC of various positively charged, pore-forming antimicrobials (i.e., CAMPs and aminoglycosides; Table 1). The human CAMP LL-37 could not be added at high enough concentrations to determine the MIC with the wild-type strain, but the decrease in the MIC after deletion of *lysX* was estimated to be >80-fold. Mutation of *lysX* decreased the MIC for the highly charged antibiotic polymyxin B by 64-fold, and for the aminoglycosides gentamicin and apramycin, the MICs decreased 64- and 32-fold, respectively. There was an apparent correlation between the degree of positive charge born by the antimicrobials and the magnitude of resistance associated with *lysX* (Table 1). Thus, *lysX* conferred the strongest protection against CAMPs with five or six positive charges (such as those listed above). Lesser effects were measured for compounds exhibiting a lower number of positive charges, and no protective advantage was observed with negatively charged antimicrobials such as dermcidin and ceftriaxone. In contrast to the *lysX* gene, deletion of *alaDAGS* did not significantly alter bacterial resistance to any of the tested antimicrobials. Therefore, we did not determine the MIC for the Δ*alaDAGS* complementation strain (Table 1).

**TABLE 1:** Minimum inhibitory concentrations against *C. pseudotuberculosis* wild-type, Δ*alaDAGS*, and Δ*lysX* mutants

Antibiotics	Charge^*a*^	MIC (µg/mL)				wt/Δ*lysX* (fold-change)
		wt	Δ*lysX*	Δ*alaDAGS*	Δ*lysX* complement	
Peptides/glycopeptides/lipopeptides						
Human LL-37^*c*^	+6	>60	0.75	>60	>60	>80
Polymyxin B	+5	640	10	640	640	64
Nisin	+4	80	2.5	80	80	32
Melittin	+5	>40	10	>40	>40	>4
Human defensin HNP-1	+3	3	0.75	3	3	4
Vancomycin	+1	0.75	0.375	0.75	N.D.^*b*^	2
Bacitracin	0	6.4	3.2	6.4	N.D.	2
Dermcidin DCD-1L	−5	>50	>50	>50	N.D.	1
Penicillins, cephalosporins						
Oxacillin	−1	2.5	1.25	2.5	N.D.	2
Ampicillin	0	1.25	1.25	1.25	N.D.	1
Azlocillin	−1	0.156	0.156	0.156	N.D.	1
Ceftriaxone	−2	0.625	0.625	0.625	N.D.	1
Macrolides						
Tulathromycin	+3	2.5	1.25	2.5	2.5	2
Erythromycin	+1	0.156	0.156	0.156	N.D.	1
Polyketides						
Tetracycline	−1	0.312	0.312	0.312	N.D.	1
Oxytetracycline	0	0.625	0.625	0.625	N.D.	1
Aminoglycosides						
Gentamicin	+5	5	0.078	5	5	64
Apramycin	+5	20	0.62	20	20	32
Streptomycin	+3	20	1.25	20	20	16
Kanamycin	+4	10	1.25	10	10	8
Other						
Spectinomycin	+1	2.5	1.25	2.5	2.5	2
Chloramphenicol	0	10	10	10	N.D.	1

^*a*^ Charge at pH 7, DrugBank (89).

^*b*^ N.D., not determined.

^*c*^ Human peptide LL-37 fragment (18–37).

### Δ*lysx* cells exhibit altered membrane permeability

**FIG 5:**
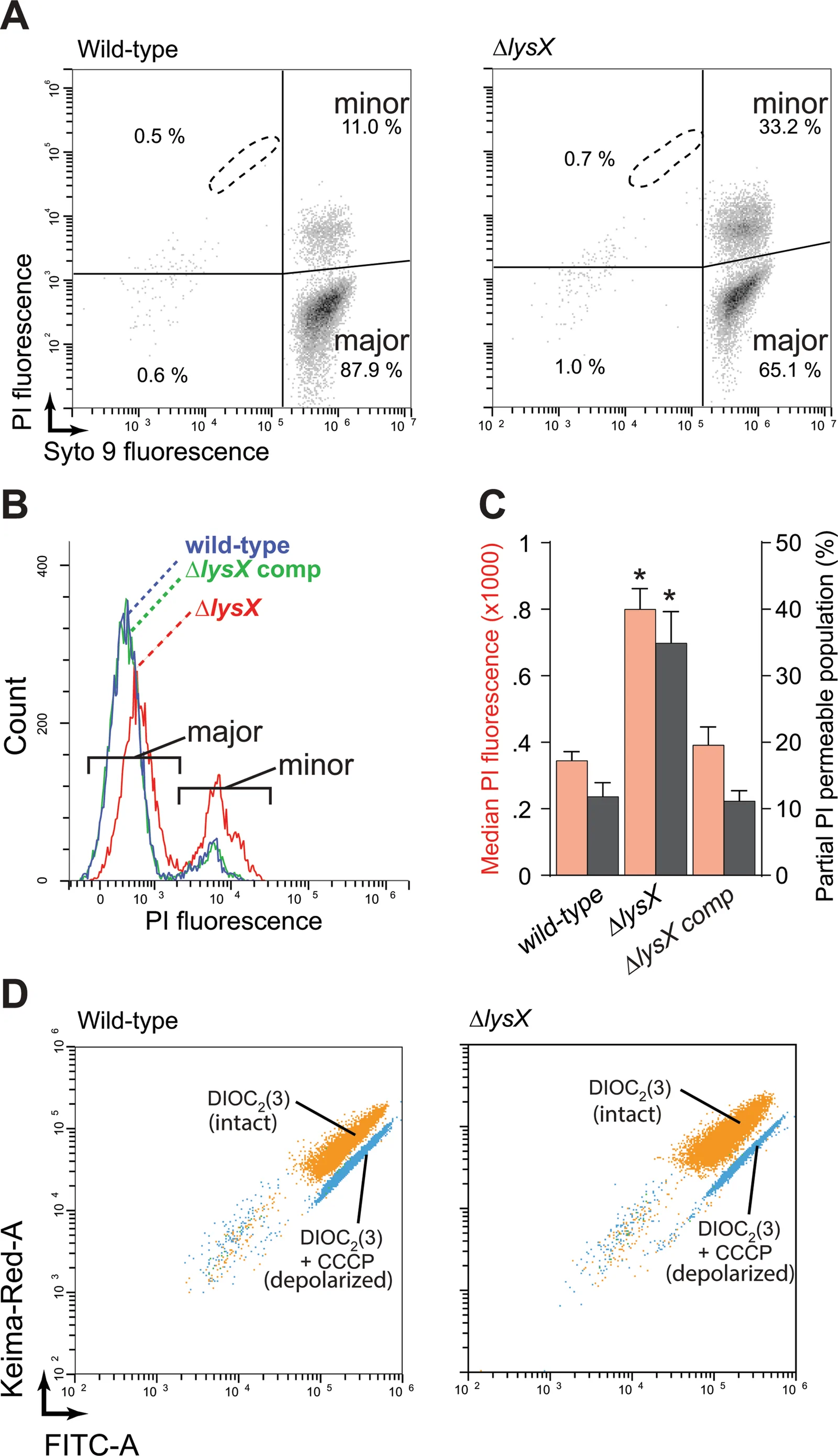
Permeability and membrane potential of *C. pseudotuberculosis* wild-type and Δ*lysX* cells. (**A**) Density plots of *C. pseudotuberculosis* stained with SYTO 9 and PI. Dashed lines demarcate the positions of dead cells used as controls. The data were gated as shown in Fig. S3. Major and minor populations of cells can be distinguished according to PI permeability. (**B**) Histogram of PI fluorescence of the cells shown in A. A shift in the PI fluorescence was observed for the Δ*lysX* population of cells. (**C**) The size of the subpopulation exhibiting partial PI permeability (data shown in A, upper right quadrant) is represented by gray bars. The median PI fluorescence of the major cell population (data shown in B, lower right quadrant) is represented by red bars. Histograms with error bars represent means ± SD’s and were derived from three independent experiments (*n* = 3). * Indicates a significant difference with the wt strain, *P* < 0.0003. (**D**) Dot plot of *C. pseudotuberculosis* stained with the membrane potential-sensitive dye DIOC_2_(3). Green (FITC-A) versus red fluorescence (Keima-Red-A) of DiOC_2_(3) was measured in control bacteria (orange dots) and bacteria depolarized with CCCP (blue dots). The data were gated as shown in Fig. S4. A decrease in the red signal after addition of CCCP demonstrates the presence of a membrane potential in each of the strains tested (49).

Because *lysX* was found to be associated with resistance to many antibiotics, the membrane integrity of Δ*lysX* cells was assessed using the *Bac*Light LIVE/DEAD Bacterial Viability assay (Invitrogen) and compared to that of the wild-type strain. This assay uses SYTO 9 and PI dyes, which both exhibit a net positive charge. When bound to cellular nucleic acids, these dyes fluoresce green and red, respectively (48). SYTO 9 is membrane-permeable and stains all bacterial cells, while PI only penetrates those exhibiting a compromised membrane. PI permeation displaces SYTO 9 from its binding sites and is often used as an indicator for dead cells. *C. pseudotuberculosis* strains were stained and analyzed by flow cytometry and compared to dead (heat-treated) bacterial cultures as a control (Fig. 5A). A cytometry gate for bacterial cells was set using forward and side scattering parameters and used for all samples (Fig. S3).

Although deletion of *lysX* negatively affected the generation time of *C. pseudotuberculosis* (Fig. S2), it did not alter cell viability. Figure 5A shows that both wild-type and Δ*lysX* strains exhibited a low level of dead cells (upper left quadrant) and two predominant subpopulations of cells with different levels of PI fluorescence (right quadrants). The major population of cells (lower right quadrant) exhibited a lower PI fluorescence signal, characteristic of live bacteria impermeable to PI. The minor population in the upper right quadrant exhibited the same SYTO 9 signal as the major population but a PI signal that was intermediate between that of PI-impermeable cells (bottom right quadrant) and control dead cells (top left quadrant), suggesting that this population exhibits partial permeability to PI. This minor population was three times larger in the Δ*lysX* strain (33.2 ± 4.7%) than in wild-type cells (11.0 ± 2.1%, *P* = 0.0003), demonstrating that *lysX* deletion increases overall permeability to PI (Fig. 5C). PI fluorescence within the major population of cells also shifted upward along the y-axis for the Δ*lysX* strain (2.3-fold higher than wild-type; Fig. 5B and C, *P* < 0.0001), demonstrating that a loss of *lysX* increases permeability of the membrane to PI within this subpopulation as well.

Cell populations exhibiting partial permeability to PI have been reported before with various bacterial species cultured in standard or challenge conditions with antimicrobials (50, 51). These populations are often referred to as injured, compromised, or damaged (50, 52). To investigate whether the Δ*lysX* strain exhibits notable membrane damage, which could explain altered membrane permeability and sensitivity to antibiotics, membrane functionality as an impermeable barrier was qualitatively assessed using the membrane potential-sensitive dye DiOC_2_(3) (Fig. 5D; Fig. S4). This dye emits green fluorescence in the monomeric state when adsorbed on the surface of cells and red fluorescence in the aggregated state after translocation inside the cell in a membrane potential-dependent manner. When cells are exposed to the proton ionophore CCCP, the proton gradient dissipates, and the membrane potential decreases, which in turn decreases DiOC_2_(3) import and the red fluorescence signal (49). Figure 5D (see Fig. S4 for details) shows that in the absence of CCCP, *C. pseudotuberculosis* wild-type and Δ*lysX* cells exhibit a similar red fluorescence signal indicative of a membrane potential. These findings suggest that, although *lysX* deletion increased permeation of PI across the membrane in a subpopulation of cells, the bacterial membrane retained its capacity to maintain a proton motive force and therefore did not show evidence of substantial mechanical damage.

### *lysX* increases ***C. pseudotuberculosis*** virulence in a ***G. mellonella*** infection model**.**

**FIG 6:**
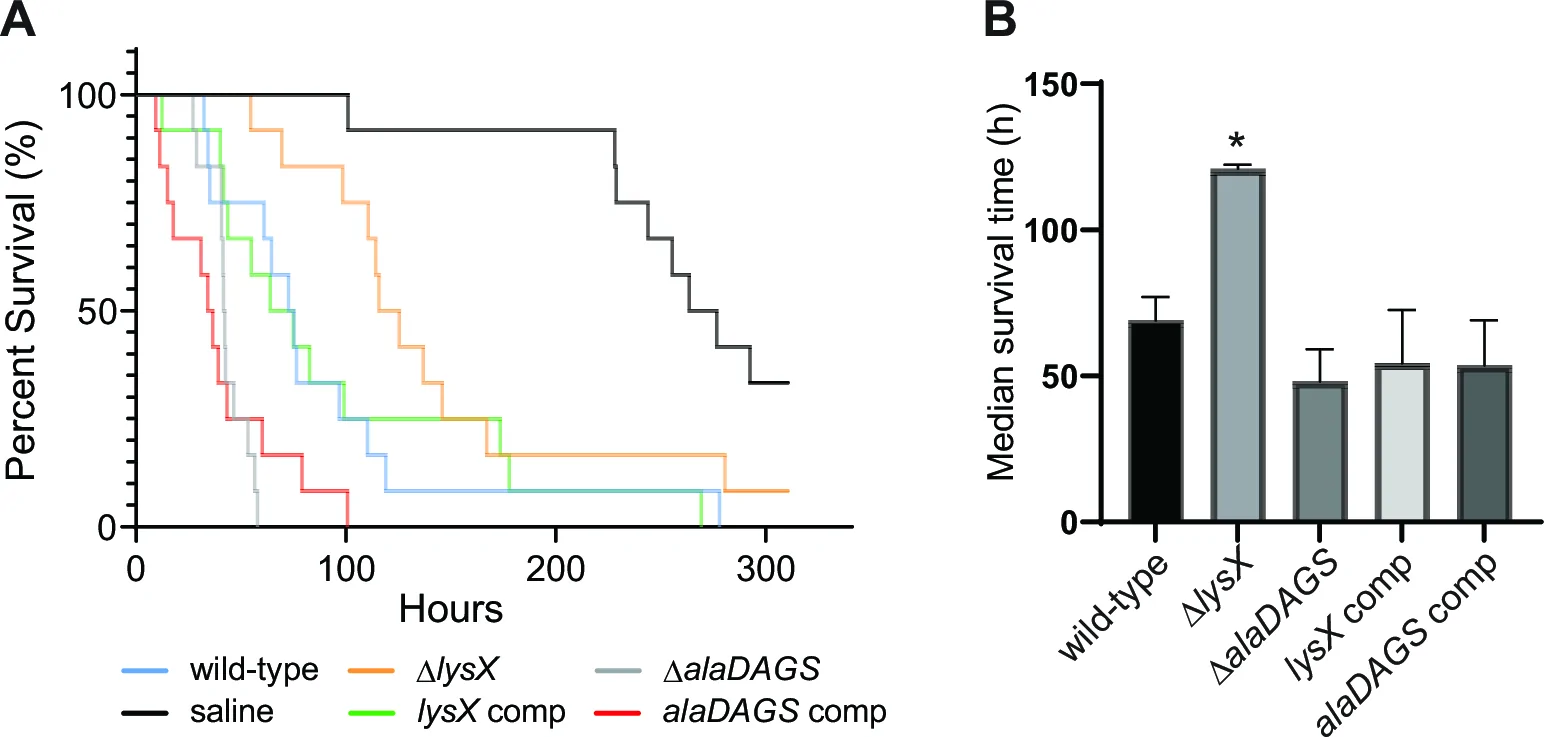
*G. mellonella* survival after infection with *C. pseudotuberculosis*. (**A**) Kaplan-Meier survival curves of infected larvae. Larvae were infected with 2.5 × 10^5^ CFU of *C. pseudotuberculosis* strains (wild-type, mutant, or complementation) as described in Materials and Methods. (**B**) Median survival time of larvae infected with *C. pseudotuberculosis* strains. Histograms with error bars represent means ± SD’s derived from four independent experiments (*n* = 4). *Indicates a significant difference compared to the wt strain, *P* = 0.006.

To determine whether *lysX* and *alaDAGS* play a role in *C. pseudotuberculosis* virulence, *G. mellonella* larvae were inoculated with the *lysX*- and *alaDAGS*-deficient strains and compared to a control group inoculated with the wild-type strain. The median survival time was determined for each group (Fig. 6). Larvae infected with the wild-type strain exhibited a median survival time of 69 ± 8 h (*n* = 4). The survival time of larvae infected with Δ*lysX* cells was increased by 75% (121 ± 1.3 h, *P* = 0.006, *n* = 4), while no significant difference was observed with larvae inoculated with the Δ*alaDAGS* mutant. These experiments support the hypothesis that *lysX* is a virulence factor in *C. pseudotuberculosis* in a *G. mellonella* infection model.

## DISCUSSION

Lys-DAG was reported in 1988 in *Mycobacterium phlei*, and its biosynthesis and biological significance have since remained unknown (34, 35). Here, we show that LysX from *C. pseudotuberculosis* and two mycobacterial species synthesizes Lys-DAG and not Lys-PG. Lys-DAG confers resistance to *C. pseudotuberculosis* against many positively charged antimicrobials, and resistance is proportional to the number of charges born by the antimicrobial. We show that Lys-DAG is a bacterial virulence factor (in *C. pseudotuberculosis*) using an insect infection model. Lys-DAG and Ala-DAG are abundant amine-containing lipids in *C. pseudotuberculosis* that is readily detected by TLC. LC-MS analysis also revealed low levels of Lys-PG *in vivo* that were not detectable by TLC analysis. The synthesis of Lys-DAG and Lys-PG both appear to be dependent on *lysX*. Heterologous expression of *lysX* in *C. glutamicum* did not yield any detectable Lys-PG, but only Lys-DAG. Reconstitution of LysX activity *in vitro* and determination of the reaction product with TLC or LC-MS only revealed the synthesis of Lys-DAG. This lipid specificity for DAG was also demonstrated for LysX orthologs originating from two *Mycobacterium* species. LC-MS analysis of lipid extracts from *M. abscessus* only revealed the presence of Lys-DAGs, and no Lys-PG was detected. Altogether, these results suggest that Lys-PG in *C. pseudotuberculosis* may be synthesized through a downstream pathway dependent on LysX, like the synthesis of Ala-PG in *C. glutamicum* (13). This would involve transferase activity to translocate Lys from Lys-DAG to PG. The presence or expression of this downstream pathway may be species-specific or may be induced under certain culture conditions. These possibilities would explain the presence of Lys-PG in *M. turberculosis* and its apparent dependence on *lysX* (20).

Similar to the role of Lys-PG in other bacterial species [for review, see references (15 – 18)], the synthesis of Lys-DAG provided *C. pseudotuberculosis* with increased resistance against various positively charged antimicrobial peptides and aminoglycosides. Δ*lysX* markedly impacted MICs, with a large effect of up to >80-fold. MIC breakpoints for antibiotics to treat *Corynebacterium* infection are scarce (53, 54) compared to those available for other more common pathogens (55). However, the susceptibility breakpoint for gentamicin reported for *Corynebacterium* spp. is 4 µg/mL (53). *C. pseudotuberculosis* wild-type and Δ*lysX* strains exhibiting MICs of 5 and 0.075 µg/mL, respectively, show that *lysX* alone could potentially affect the treatment outcomes of a *C. pseudotuberculosis* infection, which reaffirms the relevance of lipid aminoacylation systems as an important mechanism of antibiotic resistance. On the other hand, the synthesis of Ala-DAG does not affect growth, antibiotic resistance, or virulence, which corroborate earlier observations in *C. glutamicum* for this lipid (13). The biological significance of Ala-DAG remains to be uncovered.

Flow cytometry data presented here suggest that the wide-spectrum antibiotic sensitivity of the Δ*lysX* strain is the result of an alteration of the membrane permeability to cationic compounds rather than a membrane disruption. CAMPs and aminoglycosides must first ionically associate with the cytoplasmic membrane to produce their effects (56, 57), and it is likely that Lys-DAG decreases the binding of cationic compounds and their permeation through the membrane directly by electrostatic repulsion, as has been proposed for Lys-PG in other bacteria (2, 58 – 60). In addition, aminoacylated PG is known to establish salt bridges with neighboring phosphate groups, which stabilize membranes (61 – 63). Interestingly, *C. pseudotuberculosis* exhibits two populations of cells with different membrane permeabilities to PI. The size of the population of cells exhibiting high PI permeability is increased in the Δ*lysX* strain. This increased PI permeability is not the result of a compromised cell membrane because the membrane still supports membrane potential. PI permeability has been used for decades as a marker for dead cells or for cells exhibiting extensive membrane damage (64). However, various reports suggest that some bacterial species exhibit a transient and natural permeability to PI during their cell cycle or during some specific and reversible metabolic state (65, 66). The reason for this staining pattern is unknown, but microscopy and flow cytometry evidence in *Corynebacterium* and *Mycobacterium* species suggests that this phenomenon may be the result of a normal asymmetric (hyphae-like) cell division process, yielding one of the daughter cells with a transiently higher permeability to PI (65, 67). Together with the fact that the *ΔlysX* strain exhibits an increased generation time, it is possible that *lysX* may play a role in cell division in *C. pseudotuberculosis*. This hypothesis is supported by additional observations regarding the role of lipid lysylation in other bacteria. In *M. tuberculosis*, *lysX* deletion disturbs cell division (29). The septal localization of MprF (synthesizing Lys-PG) during division is also evident in several bacterial species (58, 68). In *Enterococcus faecalis*, septal localization of MprF protects negatively charged microdomains hosting the SecA/Sortase A secretory system against CAMPs during cell division (58). It has been proposed that the lack of an MprF homolog in *Streptococcus pyogenes* is responsible for its sensitivity against polymyxin B, which targets the negatively charged microdomain hosting the exportal secretory system adjacent to the septum (69). Among the CAMPs tested, polymyxin B had one of the most pronounced effects on the *C. pseudotuberculosis* Δ*lysX* mutant. It is possible that Lys-DAG may play a role similar to that of Lys-PG in other bacteria, modulating membrane affinity for CAMPs and protecting specific foci on the cell surface.

## MATERIALS AND METHODS

### Bacterial strains and growth conditions

*C. pseudotuberculosis* ATCC 19410 was obtained from the American Type Culture Collection and grown in BHI (BD Biosciences) at 37°C with aeration by shaking. Alternatively, cells were grown on plates of the same media containing 1.5% (wt/vol) agar and supplemented with 25 mg L^−1^ of kanamycin, as necessary. *M. abscessus* was grown at 37°C on solid LB or in liquid BHI or Sauton media. Liquid media were supplemented with 10% (vol/vol) Oleic Albumin Dextrose Catalase (OADC), prepared as previously described (70).

### *C. pseudotuberculosis* and *M. abscessus* competent cells and transformation

Electrocompetent *C. pseudotuberculosis* cells were prepared according to a protocol developed by Dorella et al. (71). Briefly, a starter culture, inoculated using a single colony isolated from BHI, was grown overnight at 37°C and subsequently used to inoculate 200 mL of BHI. Cells were harvested when the optical density at 600 nm (OD_600_) reached 0.8 and washed four times with decreasing volumes (100, 80, 50, and 25 mL) of ice-cold 15% (vol/vol) glycerol. Then, they were resuspended to a final OD of 60 and stored as aliquots at –80°C. Electroporation was achieved with 45 µL of competent cells and 0.5 µg of plasmid DNA in a 1 mm electroporation cuvette. Electroporation parameters were 1,900 V, 200 Ω, and 25 µF. Cells were immediately resuspended in 800 µL of BHI, incubated at 37°C for 2 h, and plated on selective BHI. Transformants were recovered after 48 h of growth at 37°C. *C. glutamicum* cells were transformed as previously described (13).

Electrocompetent cells of *M. abscessus* were prepared according to the protocol described by Viljoen et al. (45). Briefly, single colonies grown on LB solid medium were used to inoculate 5 mL of Sauton + OADC and 0.025% (vol/vol) tyloxapol. Cells were grown for 6 days at 37°C with agitation until the OD_600_ reached 5.0. Then, 1 mL fractions of the bacterial suspension were stored at −80°C. Frozen aliquots were used to inoculate 200 mL of BHI + OADC and grown for 23 h at 37°C with constant agitation (200 rpm) until the OD reached 0.8. Cultures were chilled on ice for 20 min, and cells were washed with decreasing volumes (50, 25, 25, and 5 mL) of ice-cold 10% (vol/vol) glycerol. Bacteria were resuspended in 2 mL of the wash solution (OD was typically around 70) and stored at −80°C in 200 µL of aliquots. For transformation, aliquots were thawed in the presence of 0.5 µg of plasmid DNA. Electroporation was achieved in 2 mm cuvettes at 1,900 V, 200 Ω, and 25 µF. Bacteria were recovered in 1 mL of Sauton + OADC for 4 h at 37°C with agitation and plated on selective LB agar. Transformants were collected after 7 days of incubation at 37°C.

### General cloning strategy and expression in *C. glutamicum*

Bacterial strains, plasmids, primer sequences, and gene accession numbers are reported in Tables S1 and S5. Cloning was achieved using the FastCloning strategy described in reference (72). PCR was performed using Phusion High-Fidelity DNA Polymerase (ThermoFisher). Plasmid templates were removed by digestion with *Dpn*I, and PCR products of inserts and plasmids were transformed into competent cells (DH5α) prepared using rubidium chloride as previously described (73). *C. pseudotuberculosis* genes were cloned into the expression plasmid pEKEx2-GFP (13, 74, 75). *lysX* from *M. parafinicum* was codon optimized for expression in *C. glutamicum* and cloned into pEKEx-2 (Genscript). Expression of *C. glutamicum* transformants was achieved by incubation overnight at 30°C in THB (BD Biosciences).

### In-frame deletion of *C. pseudotuberculosis*

In-frame and markerless deletion of *C. pseudotuberculosis* genes was achieved using allelic exchange by homologous recombination in a classical two-step approach with the vector pK19mobsacB (13, 76). Briefly, the upstream and downstream sequences (1 kb each) flanking the regions targeted for deletion were PCR amplified using *C. pseudotuberculosis* ATCC19410 genomic DNA as a template (Fig. S5) and stitched together by overlap PCR using primers shown in Table S1 and Fig. S5. The resulting cassettes were cloned into the suicide vector pK19mobsacB. The *C. pseudotuberculosis* genes *lysX* and *aladags* were inactivated using the vectors pK19*ΔlysX and* pK19Δ*aladags*, respectively, as previously described (13). Briefly, plasmid constructs were transformed into *C. pseudotuberculosis* ATCC19410 by electroporation, and recombinant cells were selected on BHI supplemented with 25 mg L^−1^ of kanamycin. Plasmid insertion at the expected loci was confirmed by PCR using flanking primers (Fig. S5). To obtain markerless in-frame deletions, positive colonies were passaged three times in BHI without antibiotics, before being plated on BHI containing 10% (wt/vol) sucrose to counter-select bacteria harboring the recombinant plasmid with the counter-selectable marker SacB. Sucrose-resistant, kanamycin-sensitive clones, having lost the pK19mobsacB plasmid and containing the expected deletion, were confirmed by PCR.

### LysX disruption in *M. abscessus*

The suicide vector pVT (nearly identical to pUX1) (45) and the plasmid pVT-*lysX* (used for the disruption of *lysX* in *M. abscessus*) were built using the primers shown in Table S1 and according to the map shown in Fig. S5. Upstream and downstream flanking regions of *lysX* were cloned into pVT to generate an in-frame, markerless deletion of *the gene* in *M. abscessus* as described above. The gene was inactivated after a single crossover event, leading to the insertion of the plasmid into the chromosome as described in reference (45). Positive transformants were selected on LB supplemented with 75 mg L^−1^ of kanamycin and displayed a strong red fluorescence signal due to expression of the protein tdTomato (45). Plasmid insertion at the expected locus was verified by PCR using primers flanking the targeted region (primers 1212 and 1213; Fig. S5).

### TLC analysis of lipids from *Corynebacterium* and *M. abscessus*

Lipids were prepared from biomass after the growth of *C. glutamicum* and *C. pseudotuberculosis* in 200 mL of THB overnight at 30°C or 37°C, respectively. Lipids were prepared using the Bligh-Dyer procedure (77), with modifications as described (78). For *M. abscessus*, bacteria were grown in 200 mL of LB at 37°C for 3 days (until the OD reached 3.0) and washed in 30 mM Tris-HCl, pH 8.0. Lipids were prepared according to methods previously described (79, 80), with the following modifications. Bacteria were resuspended in 2.5 mL of methanol in a glass tube and mixed with 5 mL of chloroform. The suspension was mixed by inversion and placed at 4°C overnight. The organic phase beneath the cellular debris was collected and dried. All lipid preparations (from *Corynebacterium* or *Mycobacterium*) were resuspended in chloroform:methanol (2:1), and any insoluble material was removed by centrifugation. Lipids were analyzed by TLC using a solvent system consisting of chloroform:methanol:water (14:6:1). Total lipids were visualized using iodine vapors and amine-containing lipids with ninhydrin.

### tRNA-dependent lipid aminoacylation assay

tRNA-dependent lipid aminoacylation was measured according to established methods (81). Activity was assayed using enzymes present in a membrane extract isolated from *C. pseudotuberculosis* as previously described (81). Aminoacylation reactions contained 200 mM Hepes•NaOH, pH 7.2, 60 mM KCl, 20 mM MgCl_2_, 4 mM ATP, 2 mg/mL total tRNA (from *E. coli* MRE600; Roche Applied Science), 20 µM ^14^C-L-Lys (75 Ci/mol; Perkin Elmer), 100 nM lysyl-tRNA synthetase from *E. coli*, 0.25 mg/mL membrane extract (as determined by Bradford assay; Biorad), and a mixture of lipids consisting of 2 mg/mL egg-PG and 0.5 mg/mL of DAG (Avanti Polar Lipids). Prior to adding to the reaction mixture, egg-PG and DAG were mixed, dried, and emulsified by low-power sonication in a buffer containing 100 mM Hepes, 30 mM KCl, and 10 mM MgCl_2_. After incubation of the complete reaction mixture at 37°C, lipids were extracted using the Bligh and Dyer procedure (22), dried, and resuspended in 20 µL of chloroform:methanol (2:1). Reaction products were mixed with total lipids extracted from *C. pseudotuberculosis* and spotted on an HLF silica gel TLC plate (Analtech). TLCs were developed in chloroform:methanol:water (14:6:1), and [^14^C]-lysylated lipids were visualized by phosphorimaging. For MS analysis, [^14^C]-Lys was substituted with deuterated 4,4,5,5-D_4_ L-Lys (ThermoFisher). After incubation of the reaction mixture at 37°C for 1 h, lipids were extracted in the presence of 120 mM ammonium acetate, dried, and resuspended in chloroform:methanol (2:1) before MS analysis.

### Analysis of lipids by mass spectrometry

LC-MS/MS of total lipid extracts was performed on a liquid chromatograph Surveyor Plus system (Thermo Scientific) with the autosampler connected to an LTQ-Velos linear ion trap analyzer mounted with a heated electrospray ionization probe, HESI-II (Thermo Scientific). Lipid samples extracted from *C. pseudotuberculosis* were injected onto an Ascentis Express C18 HPLC column (10 cm × 2.1 mm, 2.7 µm; Sigma-Aldrich) at a temperature of 45°C as described in reference (82), with minor modifications. Elution was performed at a flow rate of 260 µL/min with a binary gradient, where A was 60:40 water:acetonitrile and B was 90:10 isopropanol:acetonitrile. Both solutions contained 0.1% formic acid and 10 mM aqueous ammonium formate. Elution was performed over 19 min with the following gradient conditions: for the first minute, B was maintained at 32%; from 1 to 2 min, B was increased to 62%; from 2 to 9 min, B was increased to 75%; and from 9 to 16 min, B was increased to 100%. Solution B was then maintained at 100% for 3 min. The instrument was tuned with egg phosphatidylglycerol in solvent B (Avanti Polar Lipids). The drying gas flow rate was 20 U, and the temperature of the ESI was 350°C. Full-scan spectra were collected in the 110–2000 m/z range (set to positive mode), and data-dependent collision-induced dissociation tandem MS (MS2) spectra were acquired for the 15 most intense MS1 peaks in the 300–1,900 m/z range. Lipids, labeled *in vitro* with deuterated lysine, were injected directly into the electrospray interface after lipid extraction. Chromatograms were computed using the ADAP algorithm (83) and deconvoluted using the local minimum search method with MZmine 2.53 software (84). Lipids were identified using a customized version of the MS-DIAL LipidBlast library (85).

### Determination of MICs and generation time

MICs were measured in a 96-well plate using the two-fold serial microdilution method (86). Briefly, *C. pseudotuberculosis* was cultured in Mueller Hinton Broth (MHB) with 0.1% (vol/vol) Tween 80. Wells contained 150 µL of medium inoculated with 28,000 CFU of starter cells and 5 µL of inhibitory compounds (or water as blank). Growth (OD at 600 nm) was recorded in real time in an ELx808 BioTek microplate reader with constant shaking at 37°C. The MIC was defined as the lowest drug concentration inhibiting growth after 24 h of incubation. The reported MIC values represent the means of at least three independent determinations. The specific maximal growth rate (µmax) was determined using the log-linear model of the R package *growthrates* (87, 88), with data from at least 25 growth curves carried out in the absence of antibiotics. Generation times were calculated using the relationship Ln2/µmax.

### Flow cytometry

Overnight cultures of *C. pseudotuberculosis* were grown at 37°C in BHI supplemented with 0.1% (vol/vol) Tween 80. Strains were re-inoculated in BHI (without Tween) at a final OD_600_ of 0.1 and cultured at 37°C. At an OD_600_ of 0.6, cells were washed and resuspended in Dulbecco’s phosphate-buffered saline (Thermo Fisher Scientific) at a final OD_600_ of 0.16. For the *Bac*Light LIVE/DEAD Bacterial Viability assay (Invitrogen), cells were stained using 3.3 and 20 µM of Syto 9 and PI, respectively. Dead cells (for controls) were obtained by incubating bacteria at 80°C for 10 min. For the *Bac*Light Bacterial Membrane Potential assay (Invitrogen), 3,3′-diethyloxacarbocyanine iodide [DIOC_2_(3)] was added to a final concentration of 30 µM, and uncoupling was achieved using 5 µM of the protonophore m-chlorophenylhydrazone (CCCP). Treated cells were incubated for 1 h in the dark at room temperature before counting. Cells were analyzed in a CytoFLEX S Flow Cytometer (Beckman Coulter), and the data were analyzed using the software CytExpert 2.3 (Beckman Coulter). DIOC_2_(3) excitation was carried out at 488 nm, and fluorescence emission was measured at 610 (Keima red filter) and 425 nm (FITC filter). Excitation/emission wavelengths for Syto 9 and PI were 488/525 (FITC filter) and 561/610 (ECD filter), respectively.

### *Galleria mellonella* infection

*G. mellonella* larvae were purchased from Timberline Fisheries (Marion, IL, USA). Overnight starters of *C. pseudotuberculosis* were grown overnight in BHI supplemented with 0.1% (vol/vol) Tween 80. Strains were re-inoculated at a final OD_600_ of 0.1 in BHI without the addition of Tween and subsequently grown to an OD_600_ of 1.5. Bacteria were washed and resuspended in a saline solution containing 150 mM NaCl. Groups of 12 larvae weighing between 250 and 350 mg were infected with *C. pseudotuberculosis* by injection in the right foremost leg of the insect with 10 µL of saline solution containing 2.5 × 10^5^ CFU. The larvae were incubated at 37°C, and their appearance and behavior were recorded using a webcam. Survival status was checked periodically, and larvae were counted as dead when they changed color to dark brown and stopped moving. Death was confirmed by unresponsiveness to gentle manipulation. The median survival time was derived from a Kaplan-Meier survival plot using GraphPad Prism 9.1 (GraphPad Software, San Diego, CA, USA). This value was calculated as the minimum time at which 50% of the population of infected worms had died.

### Statistical analysis

Means and SDs were calculated from at least three replicates, as indicated. Means were compared using a one-way analysis of variance test with Bonferroni’s correction using GraphPad Prism 9.1. *P*-values below 0.05 were considered significant.

## SUPPLEMENTAL MATERIAL

Table S1-S2, Figure S1-S5 spectrum.01429-23-s0001.pdf Supplemental Tables and Figures.

ASM does not own the copyrights to Supplemental Material that may be linked to, or accessed through, an article. The authors have granted ASM a non-exclusive, world-wide license to publish the Supplemental Material files. Please contact the corresponding author directly for reuse.

## References

[1] Joo HS , Fu CI , Otto M . 2016. Bacterial strategies of resistance to antimicrobial peptides. Philos Trans R Soc Lond B Biol Sci 371:20150292. doi:10.1098/rstb.2015.0292 27160595 PMC4874390

[2] Peschel A , Jack RW , Otto M , Collins LV , Staubitz P , Nicholson G , Kalbacher H , Nieuwenhuizen WF , Jung G , Tarkowski A , van Kessel KP , van Strijp JA . 2001. Staphylococcus aureus resistance to human defensins and evasion of neutrophil killing via the novel virulence factor MprF is based on modification of membrane lipids with l-lysine. J Exp Med 193:1067–1076. doi:10.1084/jem.193.9.1067 11342591 PMC2193429

[3] Slavetinsky CJ , Peschel A , Ernst CM . 2012. Alanyl-phosphatidylglycerol and lysyl-phosphatidylglycerol are translocated by the same MprF flippases and have similar capacities to protect against the antibiotic daptomycin in Staphylococcus aureus. Antimicrob Agents Chemother 56:3492–3497. doi:10.1128/AAC.00370-12 22491694 PMC3393434

[4] Ernst CM , Kuhn S , Slavetinsky CJ , Krismer B , Heilbronner S , Gekeler C , Kraus D , Wagner S , Peschel A . 2015. The lipid-modifying multiple peptide resistance factor is an oligomer consisting of distinct interacting synthase and flippase subunits. mBio 6:e02340-14. doi:10.1128/mBio.02340-14 25626904 PMC4324311

[5] Arendt W , Hebecker S , Jäger S , Nimtz M , Moser J . 2012. Resistance phenotypes mediated by aminoacyl-phosphatidylglycerol synthases. J Bacteriol 194:1401–1416. doi:10.1128/JB.06576-11 22267511 PMC3294870

[6] Yakobov N , Fischer F , Mahmoudi N , Saga Y , Grube CD , Roy H , Senger B , Grob G , Tatematsu S , Yokokawa D , Mouyna I , Latgé J-P , Nakajima H , Kushiro T , Becker HD . 2020. RNA-dependent sterol aspartylation in fungi. Proc Natl Acad Sci U S A 117:14948–14957. doi:10.1073/pnas.2003266117 32541034 PMC7334510

[7] Yakobov N , Mahmoudi N , Grob G , Yokokawa D , Saga Y , Kushiro T , Worrell D , Roy H , Schaller H , Senger B , Huck L , Riera Gascon G , Becker HD , Fischer F . 2022. RNA-dependent synthesis of ergosteryl-3beta-O-glycine in ascomycota expands the diversity of steryl-amino acids. J Biol Chem 298:101657. doi:10.1016/j.jbc.2022.101657 35131263 PMC8913301

[8] Yokokawa D , Tatematsu S , Takagi R , Saga Y , Roy H , Fischer F , Becker HD , Kushiro T . 2021. Synthesis of aminoacylated ergosterols: a new lipid component of fungi. Steroids 169:108823. doi:10.1016/j.steroids.2021.108823 33713678

[9] Klein S , Lorenzo C , Hoffmann S , Walther JM , Storbeck S , Piekarski T , Tindall BJ , Wray V , Nimtz M , Moser J . 2009. Adaptation of pseudomonas aeruginosa to various conditions includes tRNA-dependent formation of alanyl-phosphatidylglycerol. Mol Microbiol 71:551–565. doi:10.1111/j.1365-2958.2008.06562.x 19087229

[10] Roy H , Ibba M . 2008. RNA-dependent lipid remodeling by bacterial multiple peptide resistance factors. Proc Natl Acad Sci U S A 105:4667–4672. doi:10.1073/pnas.0800006105 18305156 PMC2290796

[11] Thedieck K , Hain T , Mohamed W , Tindall BJ , Nimtz M , Chakraborty T , Wehland J , Jänsch L . 2006. The MprF protein is required for lysinylation of phospholipids in listerial membranes and confers resistance to cationic antimicrobial peptides (camps) on listeria monocytogenes. Mol Microbiol 62:1325–1339. doi:10.1111/j.1365-2958.2006.05452.x 17042784

[12] Peter-Katalinic J , Fischer W . 1998. Alpha-d-glucopyranosyl-, d-alanyl- and l-lysylcardiolipin from gram-positive bacteria: analysis by fast atom bombardment mass spectrometry. J Lipid Res 39:2286–2292. 9799815

[13] Smith AM , Harrison JS , Grube CD , Sheppe AEF , Sahara N , Ishii R , Nureki O , Roy H . 2015. tRNA-dependent alanylation of diacylglycerol and phosphatidylglycerol in Corynebacterium glutamicum. Mol Microbiol 98:681–693. doi:10.1111/mmi.13150 26235234 PMC4639916

[14] Grob G , Hemmerle M , Yakobov N , Mahmoudi N , Fischer F , Senger B , Becker HD . 2022. tRNA-dependent addition of amino acids to cell wall and membrane components. Biochimie 203:93–105. doi:10.1016/j.biochi.2022.09.017 36184002

[15] Bauer ME , Shafer WM . 2015. On the in vivo significance of bacterial resistance to antimicrobial peptides. Biochim Biophys Acta 1848:3101–3111. doi:10.1016/j.bbamem.2015.02.012 25701234 PMC4540701

[16] Peschel A , Sahl H-G . 2006. The co-evolution of host cationic antimicrobial peptides and microbial resistance. Nat Rev Microbiol 4:529–536. doi:10.1038/nrmicro1441 16778838

[17] Roy H . 2009. Tuning the properties of the bacterial membrane with aminoacylated phosphatidylglycerol. IUBMB Life 61:940–953. doi:10.1002/iub.240 19787708 PMC2757517

[18] Fields RN , Roy H . 2018. Deciphering the tRNA-dependent lipid aminoacylation systems in bacteria: novel components and structural advances. RNA Biol 15:480–491. doi:10.1080/15476286.2017.1356980 28816600 PMC6103681

[19] Weidenmaier C , Peschel A , Kempf VAJ , Lucindo N , Yeaman MR , Bayer AS . 2005. DltABCD- and MprF-mediated cell envelope modifications of Staphylococcus aureus confer resistance to platelet microbicidal proteins and contribute to virulence in a rabbit endocarditis model. Infect Immun 73:8033–8038. doi:10.1128/IAI.73.12.8033-8038.2005 16299297 PMC1307050

[20] Maloney E , Stankowska D , Zhang J , Fol M , Cheng Q-J , Lun S , Bishai WR , Rajagopalan M , Chatterjee D , Madiraju MV , Rubin EJ . 2009. The two-domain lysx protein of Mycobacterium tuberculosis is required for production of lysinylated phosphatidylglycerol and resistance to cationic antimicrobial peptides. PLoS Pathog 5:e1000534. doi:10.1371/journal.ppat.1000534 19649276 PMC2713425

[21] Kristian SA , Dürr M , Van Strijp JAG , Neumeister B , Peschel A . 2003. MprF-mediated lysinylation of phospholipids in Staphylococcus aureus leads to protection against oxygen-independent neutrophil killing. Infect Immun 71:546–549. doi:10.1128/IAI.71.1.546-549.2003 12496209 PMC143157

[22] Jones T , Yeaman MR , Sakoulas G , Yang S-J , Proctor RA , Sahl H-G , Schrenzel J , Xiong YQ , Bayer AS . 2008. Failures in clinical treatment of Staphylococcus aureus infection with daptomycin are associated with alterations in surface charge, membrane phospholipid asymmetry, and drug binding. Antimicrob Agents Chemother 52:269–278. doi:10.1128/AAC.00719-07 17954690 PMC2223911

[23] Samant S , Hsu F-F , Neyfakh AA , Lee H . 2009. The bacillus anthracis protein MprF is required for synthesis of lysylphosphatidylglycerols and for resistance to cationic antimicrobial peptides. J Bacteriol 191:1311–1319. doi:10.1128/JB.01345-08 19074395 PMC2631992

[24] Dare K , Shepherd J , Roy H , Seveau S , Ibba M . 2014. LysPGS formation in Listeria monocytogenes has broad roles in maintaining membrane integrity beyond antimicrobial peptide resistance. Virulence 5:534–546. doi:10.4161/viru.28359 24603093 PMC4063814

[25] Kang J , Wiedmann M , Boor KJ , Bergholz TM . 2015. VirR-mediated resistance of Listeria monocytogenes against food antimicrobials and cross-protection induced by exposure to organic acid salts. Appl Environ Microbiol 81:4553–4562. doi:10.1128/AEM.00648-15 25911485 PMC4475887

[26] Arendt W , Groenewold MK , Hebecker S , Dickschat JS , Moser J . 2013. Identification and characterization of a periplasmic aminoacyl-phosphatidylglycerol hydrolase responsible for pseudomonas aeruginosa lipid homeostasis. J Biol Chem 288:24717–24730. doi:10.1074/jbc.M113.482935 23792962 PMC3750168

[27] Sohlenkamp C , Galindo-Lagunas KA , Guan Z , Vinuesa P , Robinson S , Thomas-Oates J , Raetz CRH , Geiger O . 2007. The lipid lysyl-phosphatidylglycerol is present in membranes of rhizobium tropici CIAT899 and confers increased resistance to polymyxin B under acidic growth conditions. Mol Plant Microbe Interact 20:1421–1430. doi:10.1094/MPMI-20-11-1421 17977153

[28] Vandal OH , Roberts JA , Odaira T , Schnappinger D , Nathan CF , Ehrt S . 2009. Acid-susceptible mutants of Mycobacterium tuberculosis share hypersusceptibility to cell wall and oxidative stress and to the host environment. J Bacteriol 191:625–631. doi:10.1128/JB.00932-08 19011036 PMC2620805

[29] Maloney E , Lun S , Stankowska D , Guo H , Rajagoapalan M , Bishai WR , Madiraju MV . 2011. Alterations in phospholipid catabolism in Mycobacterium tuberculosis lysX mutant. Front Microbiol 2:19. doi:10.3389/fmicb.2011.00019 21552395 PMC3089008

[30] Fol M , Głobińska A , Stączek P , Kowalewicz-Kulbat M , Druszczyńska M , Madiraju M , Rudnicka W . 2013. The lack of L-PG production and the repercussions of it in regards to M. tuberculosis interactions with mononuclear phagocytes. Acta Microbiol Immunol Hung 60:127–144. doi:10.1556/AMicr.60.2013.2.4 23827745

[31] Kirubakar G , Schäfer H , Rickerts V , Schwarz C , Lewin A . 2020. Mutation on lysx from Mycobacterium avium hominissuis impacts the host-pathogen interaction and virulence phenotype. Virulence 11:132–144. doi:10.1080/21505594.2020.1713690 31996090 PMC6999840

[32] Baird GJ , Fontaine MC . 2007. Corynebacterium pseudotuberculosis and its role in ovine caseous lymphadenitis. J Comp Pathol 137:179–210. doi:10.1016/j.jcpa.2007.07.002 17826790

[33] Peel MM , Palmer GG , Stacpoole AM , Kerr TG . 1997. Human lymphadenitis due to Corynebacterium pseudotuberculosis: report of ten cases from Australia and review. Clin Infect Dis 24:185–191. doi:10.1093/clinids/24.2.185 9114145

[34] Lerouge P , Lebas MH , Agapakis-Caussé C , Promé JC . 1988. Isolation and structural characterization of a new non-phosphorylated lipoamino acid from Mycobacterium phlei. Chem Phys Lipids 49:161–166. doi:10.1016/0009-3084(88)90003-5 3240562

[35] Geiger O , González-Silva N , López-Lara IM , Sohlenkamp C . 2010. Amino acid-containing membrane lipids in bacteria. Prog Lipid Res 49:46–60. doi:10.1016/j.plipres.2009.08.002 19703488

[36] Hebecker S , Krausze J , Hasenkampf T , Schneider J , Groenewold M , Reichelt J , Jahn D , Heinz DW , Moser J . 2015. Structures of two bacterial resistance factors mediating tRNA-dependent aminoacylation of phosphatidylglycerol with lysine or alanine. Proc Natl Acad Sci U S A 112:10691–10696. doi:10.1073/pnas.1511167112 26261323 PMC4553816

[37] Song D , Jiao H , Liu Z . 2021. Phospholipid translocation captured in a bifunctional membrane protein MprF. Nat Commun 12:2927. doi:10.1038/s41467-021-23248-z 34006869 PMC8131360

[38] Ernst CM , Slavetinsky CJ , Kuhn S , Hauser JN , Nega M , Mishra NN , Gekeler C , Bayer AS , Peschel A . 2018. Gain-of-function mutations in the phospholipid flippase Mprf confer specific daptomycin resistance. mBio 9:e01659-18. doi:10.1128/mBio.01659-18 30563904 PMC6299216

[39] Onesti S , Desogus G , Brevet A , Chen J , Plateau P , Blanquet S , Brick P . 2000. Structural studies of lysyl-tRNA synthetase: conformational changes induced by substrate binding. Biochemistry 39:12853–12861. doi:10.1021/bi001487r 11041850

[40] Murphy RC , Axelsen PH . 2011. Mass spectrometric analysis of long-chain lipids. Mass Spectrom Rev 30:579–599. doi:10.1002/mas.20284 21656842 PMC3117083

[41] Hsu F-F , Turk J . 2009. Electrospray Ionization with low-energy collisionally activated dissociation tandem mass spectrometry of glycerophospholipids: mechanisms of fragmentation and structural characterization. J Chromatogr B Analyt Technol Biomed Life Sci 877:2673–2695. doi:10.1016/j.jchromb.2009.02.033 PMC2723218 19269264

[42] Déziel E , Lépine F , Milot S , Villemur R . 2000. Mass spectrometry monitoring of rhamnolipids from a growing culture of pseudomonas aeruginosa strain 57RP. Biochim Biophys Acta 1485:145–152. doi:10.1016/s1388-1981(00)00039-1 10832095

[43] Wiersma CJ , Belardinelli JM , Avanzi C , Angala SK , Everall I , Angala B , Kendall E , de Moura VCN , Verma D , Benoit J , Brown KP , Jones V , Malcolm KC , Strong M , Nick JA , Floto RA , Parkhill J , Ordway DJ , Davidson RM , McNeil MR , Jackson M . 2020. Cell surface remodeling of Mycobacterium abscessus under cystic fibrosis airway growth conditions. ACS Infect Dis 6:2143–2154. doi:10.1021/acsinfecdis.0c00214 32551551

[44] Sørensen PG , Cox RP , Miller M . 2008. Chlorosome lipids from chlorobium tepidum: characterization and quantification of polar lipids and wax esters. Photosynth Res 95:191–196. doi:10.1007/s11120-007-9242-5 17929193

[45] Viljoen A , Gutiérrez AV , Dupont C , Ghigo E , Kremer L . 2018. A simple and rapid gene disruption strategy in Mycobacterium abscessus: on the design and application of glycopeptidolipid mutants. Front Cell Infect Microbiol 8:69. doi:10.3389/fcimb.2018.00069 29594066 PMC5861769

[46] Komatsuzawa H , Ohta K , Fujiwara T , Choi GH , Labischinski H , Sugai M . 2001. Cloning and sequencing of the gene, fmtC, which affects oxacillin resistance in methicillin-resistant Staphylococcus aureus. FEMS Microbiol Lett 203:49–54. doi:10.1111/j.1574-6968.2001.tb10819.x 11557139

[47] Nishi H , Komatsuzawa H , Fujiwara T , McCallum N , Sugai M . 2004. Reduced content of lysyl-phosphatidylglycerol in the cytoplasmic membrane affects susceptibility to moenomycin, as well as vancomycin, gentamicin, and antimicrobial peptides, in Staphylococcus aureus. Antimicrob Agents Chemother 48:4800–4807. doi:10.1128/AAC.48.12.4800-4807.2004 15561859 PMC529239

[48] Boulos L , Prévost M , Barbeau B , Coallier J , Desjardins R . 1999. LIVE/DEAD baclight: application of a new rapid staining method for direct enumeration of viable and total bacteria in drinking water. J Microbiol Methods 37:77–86. doi:10.1016/s0167-7012(99)00048-2 10395466

[49] Shapiro HM . 2004. Estimation of membrane potential by flow cytometry. Curr Protoc Cytom Chapter 9:Unit. doi:10.1002/0471142956.cy0906s28 18770809

[50] Teixeira P , Fernandes B , Silva AM , Dias N , Azeredo J . 2019. Evaluation by flow cytometry of escherichia coli viability in lettuce after disinfection. Antibiotics (Basel) 9:14. doi:10.3390/antibiotics9010014 31906157 PMC7168219

[51] Vanhauteghem D , Audenaert K , Demeyere K , Hoogendoorn F , Janssens GPJ , Meyer E . 2019. Flow cytometry, a powerful novel tool to rapidly assess bacterial viability in metal working fluids: proof-of-principle. PLoS One 14:e0211583. doi:10.1371/journal.pone.0211583 30707728 PMC6358156

[52] Ben-Amor K , Heilig H , Smidt H , Vaughan EE , Abee T , de Vos WM . 2005. Genetic diversity of viable, injured, and dead fecal bacteria assessed by fluorescence-activated cell sorting and 16S rRNA gene analysis. Appl Environ Microbiol 71:4679–4689. doi:10.1128/AEM.71.8.4679-4689.2005 16085863 PMC1183343

[53] CLSI . 2018. Methods for antimicrobial dilution and disk susceptibility testing of infrequently isolated or fastidious bacteria. Clinical and Laboratory Standards Institute, Wayne, PA. 10.1086/510431 17173232

[54] Breakpoint tables for interpretation of MICS and zone diameters. 2022. Version 12:0. http://www.eucast.org/fileadmin/src/media/PDFs/EUCAST_files/Breakpoint_tables/Breakpoint_table_v_4.0.pdf.

[55] 2018. Performance standard for antimicrobial susceptibility testing; twenty-fifth informational supplement. Clinical and Laboratory Standards Institute, Wayne, PA.

[56] Krause KM , Serio AW , Kane TR , Connolly LE . 2016. Aminoglycosides: an overview. Cold Spring Harb Perspect Med 6:a027029. doi:10.1101/cshperspect.a027029 27252397 PMC4888811

[57] Lee T-H , Hall KN , Aguilar M-I . 2016. Antimicrobial peptide structure and mechanism of action: a focus on the role of membrane structure. Curr Top Med Chem 16:25–39. doi:10.2174/1568026615666150703121700 26139112

[58] Kandaswamy K , Liew TH , Wang CY , Huston-Warren E , Meyer-Hoffert U , Hultenby K , Schröder JM , Caparon MG , Normark S , Henriques-Normark B , Hultgren SJ , Kline KA . 2013. Focal targeting by human beta-defensin 2 disrupts localized virulence factor assembly sites in Enterococcus faecalis. Proc Natl Acad Sci U S A 110:20230–20235. doi:10.1073/pnas.1319066110 24191013 PMC3864318

[59] Mukhopadhyay K , Whitmire W , Xiong YQ , Molden J , Jones T , Peschel A , Staubitz P , Adler-Moore J , McNamara PJ , Proctor RA , Yeaman MR , Bayer AS . 2007. In vitro susceptibility of Staphylococcus aureus to thrombin-induced platelet microbicidal protein-1 (tPMP-1) is influenced by cell membrane phospholipid composition and asymmetry. Microbiology 153:1187–1197. doi:10.1099/mic.0.2006/003111-0 17379728

[60] Koprivnjak T , Peschel A , Gelb MH , Liang NS , Weiss JP . 2002. Role of charge properties of bacterial envelope in bactericidal action of human group IIA phospholipase A2 against Staphylococcus aureus. J Biol Chem 277:47636–47644. doi:10.1074/jbc.M205104200 12359734

[61] Sacré MM , El Mashak EM , Tocanne JF . 1977. A Monolayer (pi,deltaV) study of the ionic properties of Alanylphosphatidylglycerol: effects of pH and ions. Chem Phys Lipids 20:305–318. doi:10.1016/0009-3084(77)90071-8 23218

[62] Tocanne JF , Ververgaert PH , Verkleij AJ , van Deenen LL . 1974. A Monolayer and freeze-etching study of charged phospholipids. II. Ionic properties of mixtures of phosphatidylglycerol and lysylphosphatidylglycerol. Chem Phys Lipids 12:220–231. doi:10.1016/0009-3084(74)90076-0 4838050

[63] Cox E , Michalak A , Pagentine S , Seaton P , Pokorny A . 2014. Lysylated phospholipids stabilize models of bacterial lipid bilayers and protect against antimicrobial peptides. Biochim Biophys Acta 1838:2198–2204. doi:10.1016/j.bbamem.2014.04.018 24780374 PMC4118599

[64] Davey HM . 2011. Life, death, and in-between: meanings and methods in microbiology. Appl Environ Microbiol 77:5571–5576. doi:10.1128/AEM.00744-11 21705550 PMC3165249

[65] Shi L , Günther S , Hübschmann T , Wick LY , Harms H , Müller S . 2007. Limits of propidium iodide as a cell viability indicator for environmental bacteria. Cytometry A 71:592–598. doi:10.1002/cyto.a.20402 17421025

[66] Yang Y , Xiang Y , Xu M . 2015. From red to green: the propidium iodide-permeable membrane of shewanella decolorationis S12 is repairable. Sci Rep 5:18583. doi:10.1038/srep18583 26687136 PMC4685271

[67] Neumeyer A , Hübschmann T , Müller S , Frunzke J . 2013. Monitoring of population dynamics of Corynebacterium glutamicum by multiparameter flow cytometry. Microb Biotechnol 6:157–167. doi:10.1111/1751-7915.12018 23279937 PMC3917458

[68] Nishibori A , Kusaka J , Hara H , Umeda M , Matsumoto K . 2005. Phosphatidylethanolamine domains and localization of phospholipid synthases in bacillus subtilis membranes. J Bacteriol 187:2163–2174. doi:10.1128/JB.187.6.2163-2174.2005 15743965 PMC1064036

[69] Vega LA , Caparon MG . 2012. Cationic antimicrobial peptides disrupt the streptococcus pyogenes exportal. Mol Microbiol 85:1119–1132. doi:10.1111/j.1365-2958.2012.08163.x 22780862 PMC3646575

[70] Cortes MAM , Nessar R , Singh AK . 2010. Laboratory maintenance of Mycobacterium abscessus. Curr Protoc Microbiol Chapter 10:Unit 10D.1. doi:10.1002/9780471729259.mc10d01s18 20812213

[71] Dorella FA , Estevam EM , Cardoso PG , Savassi BM , Oliveira SC , Azevedo V , Miyoshi A . 2006. An improved protocol for electrotransformation of Corynebacterium pseudotuberculosis. Vet Microbiol 114:298–303. doi:10.1016/j.vetmic.2005.12.010 16442751

[72] Li C , Wen A , Shen B , Lu J , Huang Y , Chang Y . 2011. Fastcloning: a highly simplified, purification-free, sequence- and ligation-independent PCR cloning method. BMC Biotechnol 11:92. doi:10.1186/1472-6750-11-92 21992524 PMC3207894

[73] Green R , Rogers EJ . 2013. Chemical transformation of E. coli. Methods Enzymol 529:329–336. doi:10.1016/b978-0-12-418687-3.00028-8 24011059 PMC4037286

[74] Eikmanns BJ , Kleinertz E , Liebl W , Sahm H . 1991. A family of Corynebacterium glutamicum/escherichia coli shuttle vectors for cloning, controlled gene expression, and promoter probing. Gene 102:93–98. doi:10.1016/0378-1119(91)90545-m 1864513

[75] Lausberg F , Chattopadhyay AR , Heyer A , Eggeling L , Freudl R . 2012. A tetracycline inducible expression vector for Corynebacterium glutamicum allowing tightly regulable gene expression. Plasmid 68:142–147. doi:10.1016/j.plasmid.2012.05.001 22587824

[76] Schäfer A , Tauch A , Jäger W , Kalinowski J , Thierbach G , Pühler A . 1994. Small mobilizable multi-purpose cloning vectors derived from the Escherichia coli plasmids pk18 and pk19: selection of defined deletions in the chromosome of Corynebacterium glutamicum. Gene 145:69–73. doi:10.1016/0378-1119(94)90324-7 8045426

[77] BLIGH EG , DYER WJ . 1959. A rapid method of total lipid extraction and purification. Can J Biochem Physiol 37:911–917. doi:10.1139/o59-099 13671378

[78] Smith AM , Harrison JS , Sprague KM , Roy H . 2013. A conserved hydrolase responsible for the cleavage of aminoacylphosphatidylglycerol in the membrane of Enterococcus faecium. J Biol Chem 288:22768–22776. doi:10.1074/jbc.M113.484402 23793054 PMC3829361

[79] Chandramouli V , Venkitasubramanian TA . 1974. Effect of age on the lipids of mycobacteria. Indian J Chest Dis 16 Suppl:199–207. 4442929

[80] Singh P , Sinha R , Tandon R , Tyagi G , Khatri P , Chandra Shekhar Reddy L , Saini NK , Pathak R , Varma-Basil M , Prasad AK , Bose M . 2014. Revisiting a protocol for extraction of mycobacterial lipids. Int J Mycobacteriol 3:168–172. doi:10.1016/j.ijmyco.2014.07.008 26786484

[81] Roy H , Ibba M . 2009. Broad range amino acid specificity of RNA-dependent lipid remodeling by multiple peptide resistance factors. J Biol Chem 284:29677–29683. doi:10.1074/jbc.M109.046367 19734140 PMC2785599

[82] Bird SS , Marur VR , Sniatynski MJ , Greenberg HK , Kristal BS . 2011. Serum lipidomics profiling using LC-MS and high-energy collisional dissociation fragmentation: focus on triglyceride detection and characterization. Anal Chem 83:6648–6657. doi:10.1021/ac201195d 21774539 PMC3165109

[83] Myers OD , Sumner SJ , Li S , Barnes S , Du X . 2017. One step forward for reducing false positive and false negative compound identifications from mass spectrometry metabolomics data: new algorithms for constructing extracted ion chromatograms and detecting chromatographic peaks. Anal Chem 89:8696–8703. doi:10.1021/acs.analchem.7b00947 28752754

[84] Pluskal T , Castillo S , Villar-Briones A , Oresic M . 2010. Mzmine 2: modular framework for processing, visualizing, and analyzing mass spectrometry-based molecular profile data. BMC Bioinformatics 11:395. doi:10.1186/1471-2105-11-395 20650010 PMC2918584

[85] Tsugawa H , Ikeda K , Takahashi M , Satoh A , Mori Y , Uchino H , Okahashi N , Yamada Y , Tada I , Bonini P , Higashi Y , Okazaki Y , Zhou Z , Zhu ZJ , Koelmel J , Cajka T , Fiehn O , Saito K , Arita M , Arita M . 2020. A lipidome Atlas in MS-DIAL 4. Nat Biotechnol 38:1159–1163. doi:10.1038/s41587-020-0531-2 32541957

[86] CLSI . 2011. Susceptibility testing of mycobacteria, nocardiae, and other aerobic actinomycetes. Clinical and Laboratory Standards Institute, Wayne, PA, Wayne, PA. 31339680

[87] Petzoldt T . 2020. Growthrates: estimate growth rates from experimental data. Vienna, Austria. Available from: https://github.com/tpetzoldt/growthrates

[88] Anonymous . 2020. R: a language and environment for statistical computing. Vienna, Austria. Available from: https://www.R-project.org

[89] Wishart DS , Feunang YD , Guo AC , Lo EJ , Marcu A , Grant JR , Sajed T , Johnson D , Li C , Sayeeda Z , Assempour N , Iynkkaran I , Liu Y , Maciejewski A , Gale N , Wilson A , Chin L , Cummings R , Le D , Pon A , Knox C , Wilson M . 2018. Drugbank 5.0: a major update to the drugbank database for 2018. Nucleic Acids Res 46:D1074–D1082. doi:10.1093/nar/gkx1037 29126136 PMC5753335

