# Supplementary material for: The MprF homolog LysX synthesizes lysyl-diacylglycerol contributing to antibiotic resistance and virulence
Source: Microbiol Spectr. 2023 Sep 28;11(5):e01429-23. doi: 10.1128/spectrum.01429-23 (PMC10580965; doi:10.1128/spectrum.01429-23)
Supplement: Table S1-S2, Figure S1-S5 — Supplemental Tables and Figures. [file spectrum.01429-23-s0001.pdf]

Supporting Information for

MprF homolog LysX synthesizes lysyl-diacylglycerol which contributes to antibiotics resistance and virulence

Cameron P. Gill, Christopher Phan, Vivien Platt, Danielle Worrell, Thomas Andl, Hervé Roy

Table S1: Primers

Primer[a]	Target[b,c]	Sequence (5' to 3')	Purpose[c]
1504	*lysX*	gaaggagatataccatgttgcatattatcgattgcatg	Cloning of *lysX* in pet33b
1505	*lysX*	caccagtcatgctagccatttaacaaccccgagctgatcc	Cloning of *lysX* in pet33b
1506	pET33b	catggtatatctccttcttaaagttaa	pET33b amplification
1507	pET33b	ggctagcatgactggtgg	pET33b amplification
1180	cps *lysX*	tgcagaaggagatatagatatgttgcatattatcgattgcatg	Cloning of cps *lysX* in pEKEx2
1189	cps *lysX*	attgtgtcgagctagcttaacaaccccgagctgatcc	Cloning of cps *lysX* in pEKEx2
1182	*aladags*	tgcagaaggagatatagatatgtcaccgaccacgggcg	Cloning of cps *aladags* in pEKEx2
1185	*aladags*	attgtgtcgagctagcttatggcattcgctgtgctcgtac	Cloning of cps *aladags* in pEKEx2
1208	cps *lysX*	ccatgattacgccaagcttcctcctgtacctggtatatcgc	Generation of pK19Δ*lysX* (*cps*)
1209	cps *lysX*	ggatgttttgagtggctaactgtgctgtaaagagg	Generation of pK19Δ*lysX* (*cps*)
1210	cps *lysX*	cacagttagccactcaaaacatccgcgaagttctct	Generation of pK19Δ*lysX* (*cps*)
1211	cps *lysX*	gtcgacgcatgctctagagtgctgccacgatcttggac	Generation of pK19Δ*lysX* (*cps*)
1212	cps *lysX*	gagcctaatatcgcccctgag	Flanking site of the Δ*lysX* (*cps*) deletion
1213	cps *lysX*	cgtgtttgtcatcgctggtg	Flanking site of the Δ*lysX* (*cps*) deletion
1976	pVV16	ctcactagtgacgtagttaactagcgtac	Generation of pVT
1977	pVV16	atccactaggggctcacgcgttgcgc	Generation of pVT
1978	pTEC27	gtgagcccctagtggatctgacccgc	Generation of pVT
1979	pTEC27	taactacgtcactagtgaggtctgcctcg	Generation of pVT
1980	pVT	gctagcacaccagacaagtt	Generation of pVT-lysXmab
1985	pVT	cacctgttcctgtacggc	Generation of pVT-lysXmab
1987	*lysX* locus	actcaaactcgcagggaac	Flanking sites of pVT-lysXmab insertion
1988	*lysX* locus	ttcacattggagcgatataac	Flanking sites of pVT-lysXmab insertion
1981	mab *lysX*	ttgtctggtgtgctagcgacatccatcaaaccggca	Generation of pVT-lysXmab
1982	mab *lysX*	ctgaccatcagtgaggggggcagaccatc	Generation of pVT-lysXmab
1983	mab *lysX*	gccccctcactgatggtcagtgccgatccca	Generation of pVT-lysXmab
1984	mab *lysX*	aactacgtcactagtgaggccacagtcgaagtaccg	Generation of pVT-lysXmab
1258	*aladags*	ccatgattacgccaagcttaccaatcatccagattcttatggc	Generation of pK19Δ*aladags*
1259	*aladags*	ctaatgcctctcgacccagaaaacagcactagagtaaag	Generation of pK19Δ*aladags*
1260	*aladags*	gttttctgggtcgagaggcattagcacagtgtggacatc	Generation of pK19Δ*aladags*
1261	*aladags*	gtcgacgcatgctctagattgtaccccaagagcataaaggatt	Generation of pK19Δ*aladags*
1266	*aladags*	tggactatcggcggattaagc	Flanking site of the Δ*aladags* deletion
1267	*aladags*	gcctacagaggtggcgtcac	Flanking site of the Δ*aladags* deletion
1885	pEKEx2	catatctatatctccttctgcag	pEKEx2 amplification
2110	pEKEx2	taagctagctcgacacaatctg	pEKEx2 amplification
1897	pK19mobSacB	gcttggcgtaatcatggtca	pK19 amplification
1898	pK19mobSacB	ctagagcatgcgtcgacca	pK19 amplification

[a] Primer numbers refer to those shown on the construction maps in **Fig. S1.**

[b] Accession numbers for *lysX* and *aladags* for the genome assemblies accessible through the American Type Culture Collection (strain *C. pseudotuberculosis* ATCC19410) are BHLNLCGM_01988 and BHLNLCGM_01017, respectively. Identical sequences in the strain *C. pseudotuberculosis* C231 are viewable under Genbank accession numbers ADL11372 and ADL10459. Gene locus tags for *lysX* from *M. abscessus* 19977 and *M. parafinicum* M11 are MAB_2319c and BRW65_18575, respectively.

[c]cps: *Corynebacterium pseudotuberculosis,* mab: *Mycobacterium abscessus*

Table S2: Strains and plasmids used in this study.

Bacterial strains and plasmids[a]	Notes or genotype[b,c]	Reference or source
E. coli		
C41R	F_ *ompT gal dcm hsdS*B(rB_ mB_)(DE3) transformed with the plasmid pRARE2 (Cam^R)	(1)
DH5α	*hsdR17 recA1 gyrA endA1 relA1*	(2)
LysPGS	C41R transformed with pet33b-MprF2. Expresses LysPGS from *C. perfringens*	(3)
Corynebacterium glutamicum		
C.glutamicum ATCC13032	Wild-type	ATCC[$]
C.glutamicumΔpesTΔaladags	strain ATCC13032 exhibiting in-frame deletions of alanyl-diacylglygerol synthase (*AlaDAGS*, CAF19673) and a putative ala-DAG/PG transferase (*pesT,* CAF19674)	(4)
C.glutamicum ΔpesT Δaladags pEKEx2-*lysX*	Expresses LysX from *C. pseudotuberculosis*	This work
C.glutamicum ΔpesT Δaladags pEKEx2-*lysX Mpa*	Expresses LysX from *M. parafinicum*	This work
Corynebacterium pseudotuberculosis		
C.pseudotuberculosis ATCC19410	Wild-type strain	ATCC
C.pseudotuberculosis ΔlysX	In-frame deletion of *lysX in strain* ATCC19410	This study
C.pseudotuberculosis Δaladags	In-frame deletion of *aladags in strain* ATCC19410	This study
C.pseudotuberculosis ΔlysX comp	*C. pseudotuberculosis ΔlysX* in which *lysX* was restored using *pK19lysX*	This study
Mycobacterium abscessus		
M.abscessus ATCC 19977	Wild-type strain	ATCC
M. abscessus lysX::pVT	lysX disrupted by the vector pVT	This study
**Plasmids**		
pET33b	Expression in *E. coli* , kan^r	Novagen
pEKEx-2-GFP	*E. coli - Corynebacterium* shuttle vector. *Ptac,* gfp, kan^r	(5, 6)
pK19mobSacB	Suicide plasmid for in-frame deletion	(7)
pET33b-MprF2	Expression of MprF2 (LysPGS) from *C. perfringens*	(3)
pK19Δ*lysX*	pK19mobSacB for in-frame deletion of *lysX* in strain ATCC19410	This study
pK19*lysX*	pK19mobSacB exhibiting *lysX* to restore expression in *C. pseudotuberculosis ΔlysX*	This study
pK19Δ*aladags*	pK19mobSacB for in-frame deletion of *aladags* in strain ATCC19410	This study
pK19*aladags*	pK19mobSacB for in-frame deletion of *aladags* in strain ATCC19410	This study
pEKEx2-*lysX*	Expression of *C. pseudotuberculosis lysX* in *C. glutamicum*	This study
pEKEx2-*alaDAGS*	Expression of *C. pseudotuberculosis alaDAGS* in *C. glutamicum*	This study
pEKEx2-*lysXmpa*	Expression of *M. parafinicum lysX* in *C. glutamicum*	This study
pVV16	*E. coli* – Mycobacterium shuttle plasmid for expression in Mycobacterium. kanR	(8)
pTEC27	HygR, expresses tdTomato	Addgene, (9)
pVT	Plasmid nearly identical to plasmid pUX1(10) for gene disruption in *M. abscessus*. pVT is a recombinant of plasmids pTEC27 and pVV16. Constitutively expresses tdTomato. hygR, kanR in *M. abscessus.*	This study, (10)
pVT-*LysXmab*	Plasmid for disruption of lysX in *M. abscessus*	This study

[a] plasmid maps are shown in Fig. S1

[b] kan^r, kanamycin resistance, cam^r, chloramphenicol resistance

[c] accessions numbers for *lysX* and *aladags* for the genome assembly accessible at the American Type Culture Collection (strain *C. pseudotuberculosis* ATCC19410) are BHLNLCGM_01988 and BHLNLCGM_01017, respectively. Identical sequences in the strain *C. pseudotuberculosis* C231 are accessible under Genbank accession ADL11372 and ADL10459. Gene locus tags of lysX from *M. abscessus* 19977 and *M. parafinicum* M11 are MAB_2319c and BRW65_18575, respectively.

[c] cps: *Corynebacterium pseudotuberculosis,* mab: *Mycobacterium abscessus*

[$] ATCC: the American Type Culture Collection (https://www.atcc.org/)

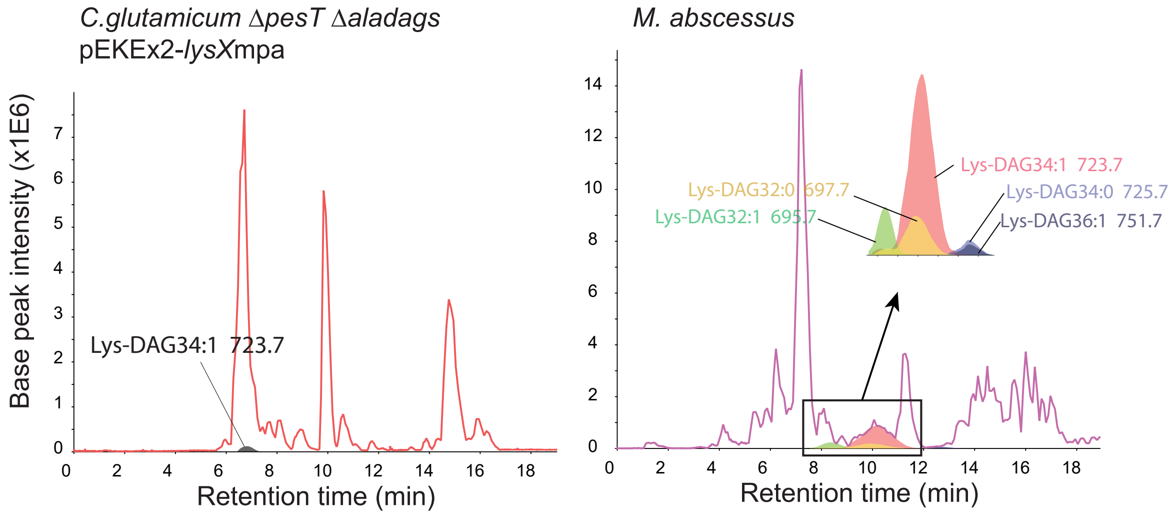

Figure S1: LC-MS/MS analysis of the product of LysX from *M. parafinicum* and *M. abscessus*. Base peak (m/z 200-2000) chromatogram of total lipids extracted from *C. glutamicum* Δ*pesT*Δ*alaDAGS* expressing *lysX* from *M. parafinicum* (pEKEx2-*lysX*mpa, left). Base peak chromatogram of total lipids extracted from wild-type *M. abscessus* (right). Aminoacylated lipids were identified using the MS2 fragmentation pattern shown in **Fig. 3**. Chromatographic deconvolution of the various species of aminoacylated lipids are shown in the inset.

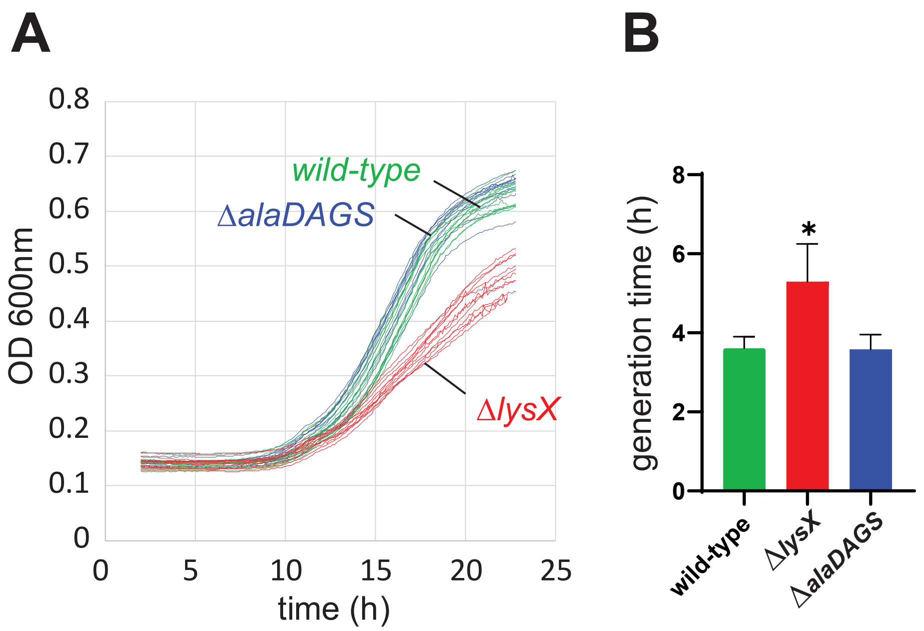

Figure S2: Generation time of *C. pseudotuberculosis*. A. Representative growth curves of wild-type *C. pseudotuberculosis*, and the Δ*lysX* and Δ*alaDAGS* strains. Bacteria were grown in MHB at 37 °C in 96-well plates as described in Materials and Methods. **B**. Generation time of the *C. pseudotuberculosis* strains. Means and standard deviations were calculated from at least 25 replicates. *Indicates significant difference relative to the wild-type strain, $p<0.0001$. Generation times for the wild-type, Δ*lysX*, and Δ*alaDAGS* strains were 3.61±0.29, 5.29±0.95, and 3.58±0.37 h, respectively.

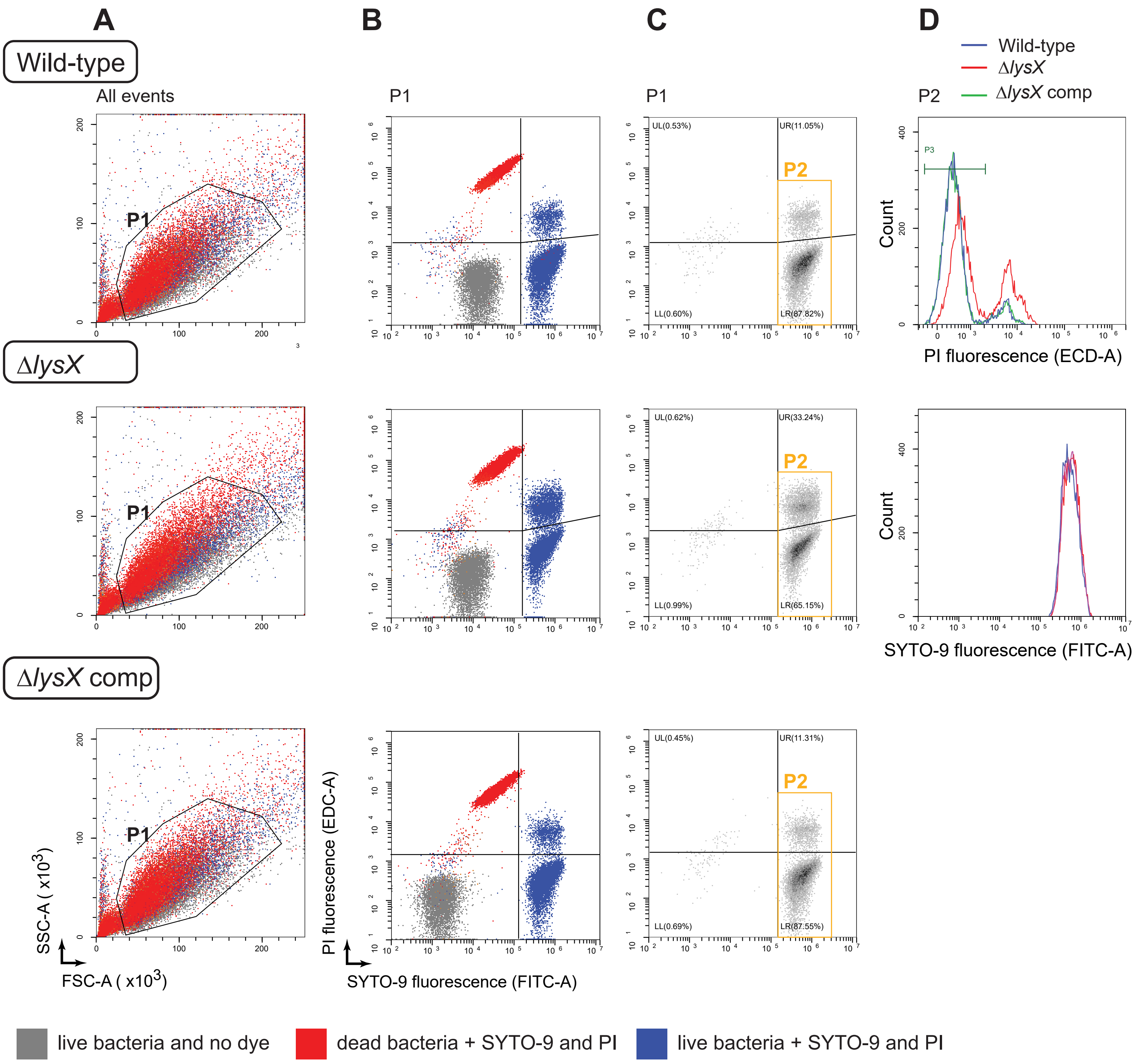

Figure S3: Flow cytometry of *C. pseudotuberculosis* stained with SYTO-9 and PI. A. Cellular populations were gated (P1) using forward and side scattering data (FSC-A, SSC-A) for each sample. **B.** Superimposed dot blots from three experiments: dyed living cells (blue), dyed dead cells (red), and non-dyed living cells (gray). A population partially permeable to PI (upper right quadrant) is distinct from the dead population of cells (upper left quadrant). **C.** Density blot plot of dyed living cells as displayed in **Fig. 5**. This data set is a representative example out of three independent experiments. **D.** Histograms of PI (as displayed in **Fig. 5**) and SYTO-9 fluorescence of the cells shown in C. A shift of the PI fluorescence was observed for the Δ*lysX* population of cells. Strains stained equally with Syto-9.

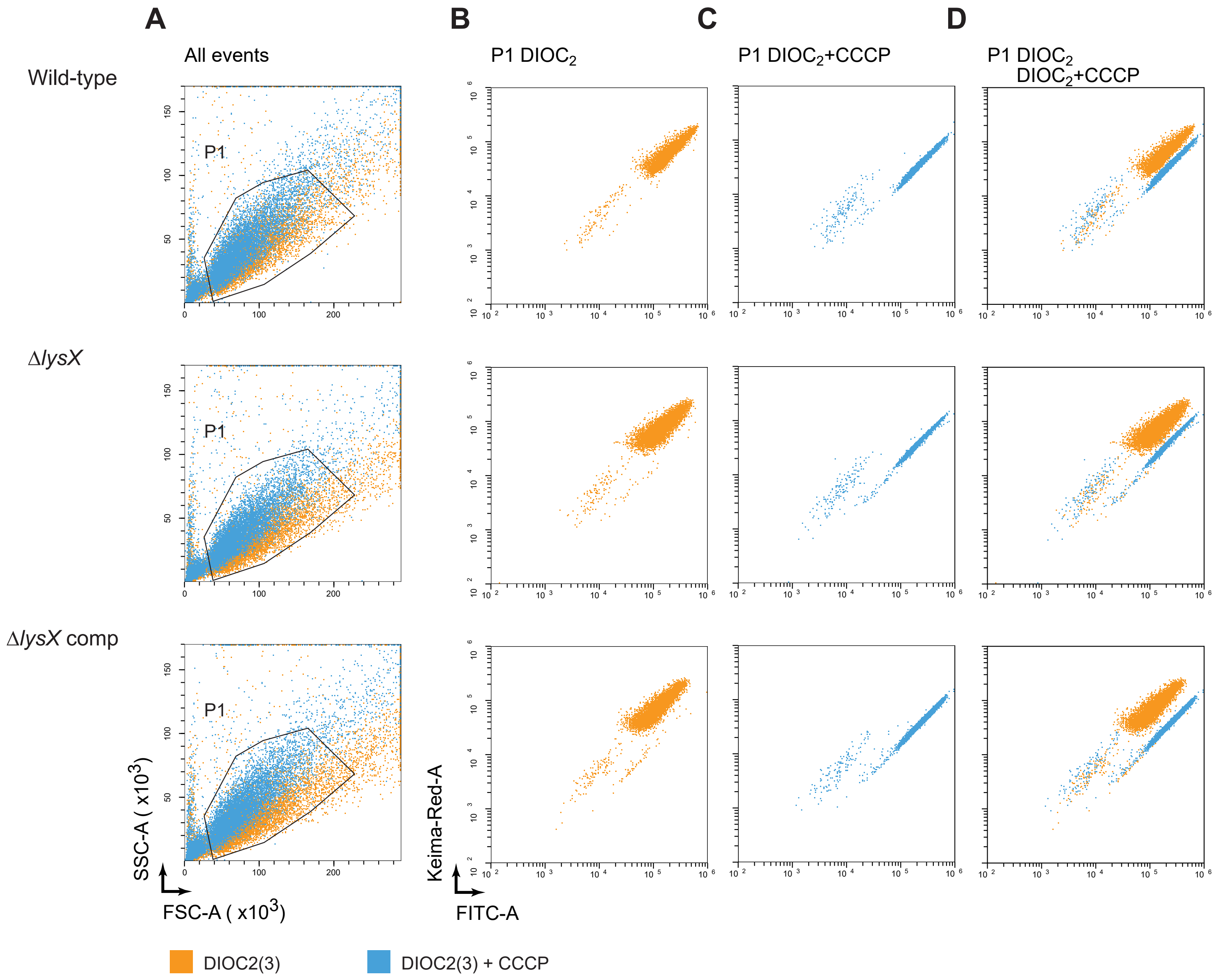

Figure S4: Membrane potential in *C. pseudotuberculosis* wildtype and Δ*lysX*. A. Cells were gated (P1) using forward and side scattering data (FSC-A vs. SSC-A) for each sample. *C. pseudotuberculosis* was stained with the membrane potential-sensitive probe DIOC2(3). Green (FITC-A) and red fluorescence (Keima-Red-A) of DiOC2(3) was measured in control bacteria (**B**) and in cells depolarized with CCCP (**C**). **D** Superimposition of data shown in B and C (also shown in **Fig. 5)**.

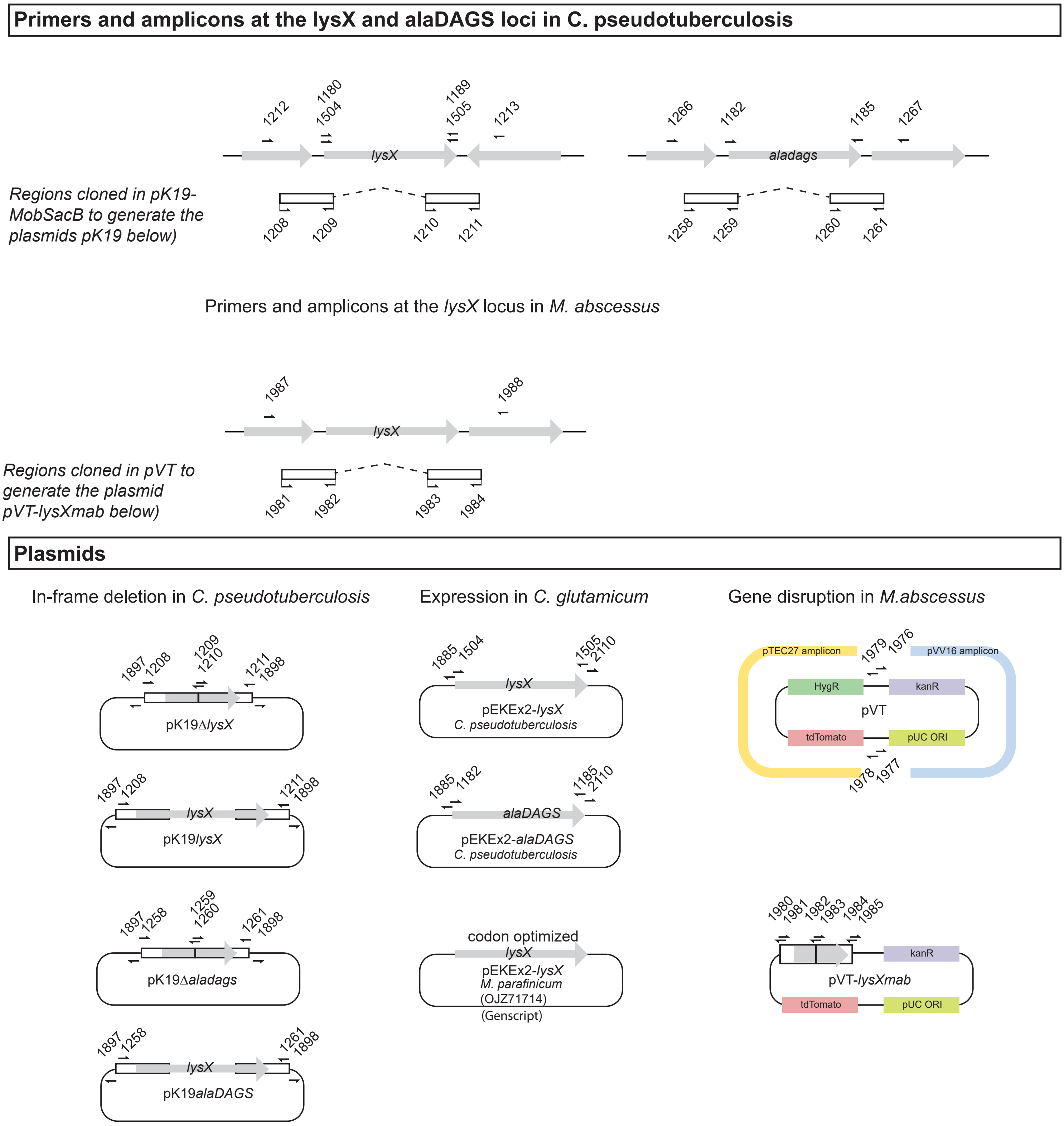

Figure S5: primers and plasmids used in this study. Plasmids and primers sequence are shown in Table S1.

SI References

1. B. Miroux, J. E. Walker, Over-production of proteins in *Escherichia coli*: mutant hosts that allow synthesis of some membrane proteins and globular proteins at high levels. *J Mol Biol* **260**, 289-298 (1996).
2. D. Hanahan, M. Meselson, Plasmid screening at high colony density. *Methods Enzymol* **100**, 333-342 (1983).
3. H. Roy, M. Ibba, RNA-dependent lipid remodeling by bacterial multiple peptide resistance factors. *Proc Natl Acad Sci U S A* **105**, 4667-4672 (2008).
4. A. M. Smith *et al.*, tRNA-dependent alanylation of diacylglycerol and phosphatidylglycerol in *Corynebacterium glutamicum*. *Mol Microbiol* **98**, 681-693 (2015).
5. B. J. Eikmanns, E. Kleinertz, W. Liebl, H. Sahm, A family of *Corynebacterium glutamicum*/*Escherichia coli* shuttle vectors for cloning, controlled gene expression, and promoter probing. *Gene* **102**, 93-98 (1991).
6. F. Lausberg, A. R. Chattopadhyay, A. Heyer, L. Eggeling, R. Freudl, A tetracycline inducible expression vector for *Corynebacterium glutamicum* allowing tightly regulable gene expression. *Plasmid* **68**, 142-147 (2012).
7. A. Schäfer *et al.*, Small mobilizable multi-purpose cloning vectors derived from the *Escherichia coli* plasmids pK18 and pK19: selection of defined deletions in the chromosome of *Corynebacterium glutamicum*. *Gene* **145**, 69-73 (1994).
8. A. Parikh *et al.*, Development of a new generation of vectors for gene expression, gene replacement, and protein-protein interaction studies in mycobacteria. *Appl Environ Microbiol* **79**, 1718-1729 (2013).
9. K. Takaki, J. M. Davis, K. Winglee, L. Ramakrishnan, Evaluation of the pathogenesis and treatment of Mycobacterium marinum infection in zebrafish. *Nat Protoc* **8**, 1114-1124 (2013).
10. A. Viljoen, A. V. Gutierrez, C. Dupont, E. Ghigo, L. Kremer, A Simple and Rapid Gene Disruption Strategy in Mycobacterium abscessus: On the Design and Application of Glycopeptidolipid Mutants. *Frontiers in cellular and infection microbiology* **8**, 69 (2018).
